# Novel synthetic opioids – toxicological aspects and analysis

**DOI:** 10.1080/20961790.2019.1588933

**Published:** 2019-07-03

**Authors:** Inês Tabarra, Sofia Soares, Tiago Rosado, Joana Gonçalves, Ângelo Luís, Sara Malaca, Mário Barroso, Thomas Keller, José Restolho, Eugenia Gallardo

**Affiliations:** a Centro de Investigação em Ciências da Saúde (CICS-UBI), Universidade da Beira Interior, Covilhã, Portugal;; b Laboratório de Fármaco-Toxicologia - UBIMedical, Universidade da Beira Interior, Covilhã, Portugal;; c Serviço de Química e Toxicologia Forenses, Instituto Nacional de Medicina Legal e Ciências Forenses, Delegação do Sul, Lisboa, Portugal;; d Departament of Toxicology, Institute of Forensic Medicine, Salzburg, Austria;; e nal von minden GmbH, Regensburg, Germany

**Keywords:** Forensic sciences, forensic toxicology, new synthetic opioids, biological specimens, toxicity, analysis

## Abstract

Over the past few years, there has been an emerging number of new psychoactive drugs. These drugs are frequently mentioned as “legal highs”, “herbal highs”, “bath salts” and “research chemicals”. They are mostly sold and advertised on online forums and on the dark web. The emerging new psychoactive substances are designed to mimic the effects of psychoactive groups, which are often abused drugs. Novel synthetic opioids are a new trend in this context and represent an alarming threat to public health. Given the wide number of fatalities related to these compounds reported within the last few years, it is an important task to accurately identify these compounds in biologic matrices in order to administer an effective treatment and reverse the respiratory depression caused by opioid related substances. Clinicians dealing with fentanyl intoxication cases should consider that it could, in fact, be a fentanyl analogue. For this reason, it is a helpful recommendation to include synthetic opioids in the routine toxicological screening procedures, including analysis in alternative matrices, if available, to investigate poly-drug use and possible tolerance to opioids. To address this public health problem, better international collaboration, effective legislation, effective investigation, control of suspicious “research chemicals” online forums and continuous community alertness are required. This article aims to review diverse reported fatalities associated with new synthetic opioids describing them in terms of pharmacology, metabolism, posology, available forms, as well as their toxic effects, highlighting the sample procedures and analytical techniques available for their detection and quantification in biological matrices.

## Introduction

The *Papaver somniferum*, or opium poppy, is the plant from which opium can be obtained, as its resin. Opium poppy medicinal effects have been known since the early men, and pharmacologic formulations were being sold in the mid-1800s, although the active substance of opium had not been identified yet. The isolation of opium's active ingredient, the alkaloid morphine, was published for the first time in 1805 by the German apothecary Friedrich Wilhelm Adam Sertürner [1]. Opioids are chemical substances that can bind to opioid receptors. Endogenously, human body produces opioid-like substances, which are also capable of binding to these receptors, namely, encephalins. When an opioid binds to its receptor, a mechanism involving the inhibition of cAMP is induced due the activation of G-protein coupled to these receptors, leading to opioid's known effects: analgesia, miosis, respiratory depression, sedation, constipation and a significant sense of euphoria [2]. Given this sense of euphoria and well-being, opioid users often tend to overuse them. Opioids have been one of the therapeutic groups, whose chemical structure has been illegally modified, leading to the so-called novel synthetic opioids (NSO).

Over the past few years, the number of emerging new psychoactive drugs has increased. According to the United Nations Office on Drugs and Crime (UNODC), the term New Psychoactive Substances (NPS) refers to “substances of abuse, either in a pure form or a preparation, that are not controlled by the 1961 Single Convention on Narcotic Drugs or the 1971 Convention on Psychotropic Substances, but which may pose a public health threat”. NPS are not necessarily recent synthesized, but its use on the market is recent. These drugs are frequently mentioned as “legal highs”, “herbal highs”, “bath salts” and “research chemicals” [3]. They are often formulated and sold online as “water pipe cleaners”, “mystical incense”, “dietary supplements”, “bath salts”, “collector's items” or “fertilizers for plants”. Although these products usually bring the warning “not suitable for human consumption”, in many websites it is possible to find a description of the dosage form, posology, administration method, possible complications and expected effects, which suggests the purpose for human use. The term *psychonaut* which is referred to individuals who use “entheogens to explore their psyche” has been substituted for the term *e-psychonaut* to emphasize the importance of the network in the acquisition of psychoactive substances, as well as the information about how to use them [4].

A study regarding people's motivations to use NPS using an online questionnaire on the international drug discussion forum www.bluelight.org, provided 1 551 reports of NPS use described by 619 participants between November 2014 and February 2015 and concluded that individuals claim to use synthetic opioids mainly because of the “pleasure and enjoyment” they experienced, followed by “coping with life challenges” and because of the addictive character of these substances. Although the participants of this study were mainly young males, a broad range of ages up to 75 years of both genders (16% females and 84% males) was identified [5]. Also, individuals on opioid medication seem to be substituting it with the new synthetic opioids, on a growing trend [6].

According to the World Drug Report 2016 of the UNODC, a total of 644 NPS were reported between 2008 and 2015 by several countries to the UNODC early warning advisory system on NPS. By December 2015, Europe had the larger number of countries reporting the appearing of NPS. In 2014, NPS seizures reached 34 tons, showing an increasing tendency. The first group to be target of notification was the synthetic cannabinoids, between 2012 and 2014, followed by synthetic cathinones and other substances including synthetic opioids, in 2015. NSO represented 2% of the NPS, up to 2015 [7], whereas by the end of 2016 this percentage was 4% [8]. In the European Drug Report 2017 from European Monitoring Center for Drugs and Drug Addiction (EMCDDA), it is evidenced that they were detained almost 2 L of synthetic opioids in 2015, showing an increase of 240 mL over the previous year. Of these, 85% are represented by fentanyl analogues [9].

Given the growing awareness about NPS market, a legal response was required, as these drugs were not documented in the Conventions of 1961 or 1971, their legal situation has been up to each country [3]. The European Union (EU) published on 21 November 2017 a new legislation regarding NPS, which provides a stronger EU Early Warning System (EWS), a regulation considering “information exchange on, and an early-warning system and risk-assessment procedure for, new psychoactive substances” [10] and a directive “including new psychoactive substances in the definition of ‘drug’” [11,12]. The new legislation maintains the three-step method to manage NPS – early warning, risk assessment and control measures – and hopes to strengthen the response to the emerging NPS [12].

The emerging NPS are designed to mimic the effects of psychoactive therapeutic groups, which are often abused drugs. NSO are a new trend in this context and represent an alarming threat to public health. In this group are included high-potency analogues of fentanyl such as acetylfentanyl, butyrylfentanyl (or butyrfentanyl), carfentanil, alfentanil, α-methylfentanyl, β-hydroxythiofentanyl, *cis*-3-methylfentanyl, *trans*-3-methylfentanyl, 4-chloroisobutyrylfentanyl, 4-fluorofentanyl (or *para*-fluorofentanyl), 4-fluorobutyrylfentanyl (or *para*-fluorobutyrfentanyl), 4-fluoroisobutyrylfentanyl (or *para*-fluoroisobutyrylfentanyl), 3-methylfentanyl, remifentanil sufentanil, *trans*-3-methylfentanyl, 2,2′-difluorofentanyl and furanylfentanyl, as well as non-fentanyl analogues like U-47700, AH-7921, U-49900, U-50488 and MT-45. These substances are synthesized in Asian laboratories and marketed via the Internet. NSO are marketed not only as stand-alone products but also as adulterants in heroin packages and as counterfeit opioid medications [13].

## Research methodology

This bibliographic search was performed on the PubMed database, using the following search strings: “novel synthetic opioid”, “new synthetic opioid”, “novel psychoactive substances” combined with Boolean operators, as well as, the name of each NSO described in this article combined with the term “synthetic opioid”. This search occurred in the between December 2017 and July 2018. Only articles written in English were considered. In order to assess their relevance in the context of this review, all articles fulfilling the search strings were screened independently by four of the authors. Only those papers that have been selected by at least two authors were subjected to review and were included in the manuscript.

## Results

To facilitate the reading and comprehension of this review, initially, the main NSO will be described namely their chemical structure, posology, available forms, pharmacology, metabolism, their toxic effects and information about fatalities. Afterwards, the analytical aspects associated with the determination of these drugs of abuse will be discussed. Once there are no reviews on the sample preparation techniques used to determine these drugs of abuse and considering that the greater volume of laboratory work is related to sample preparation, we have conducted a critical review of the approaches and recent trends available in laboratories to detect and quantify these compounds in biological specimens.

### Fentanyl and its analogues

**Figure 1.**
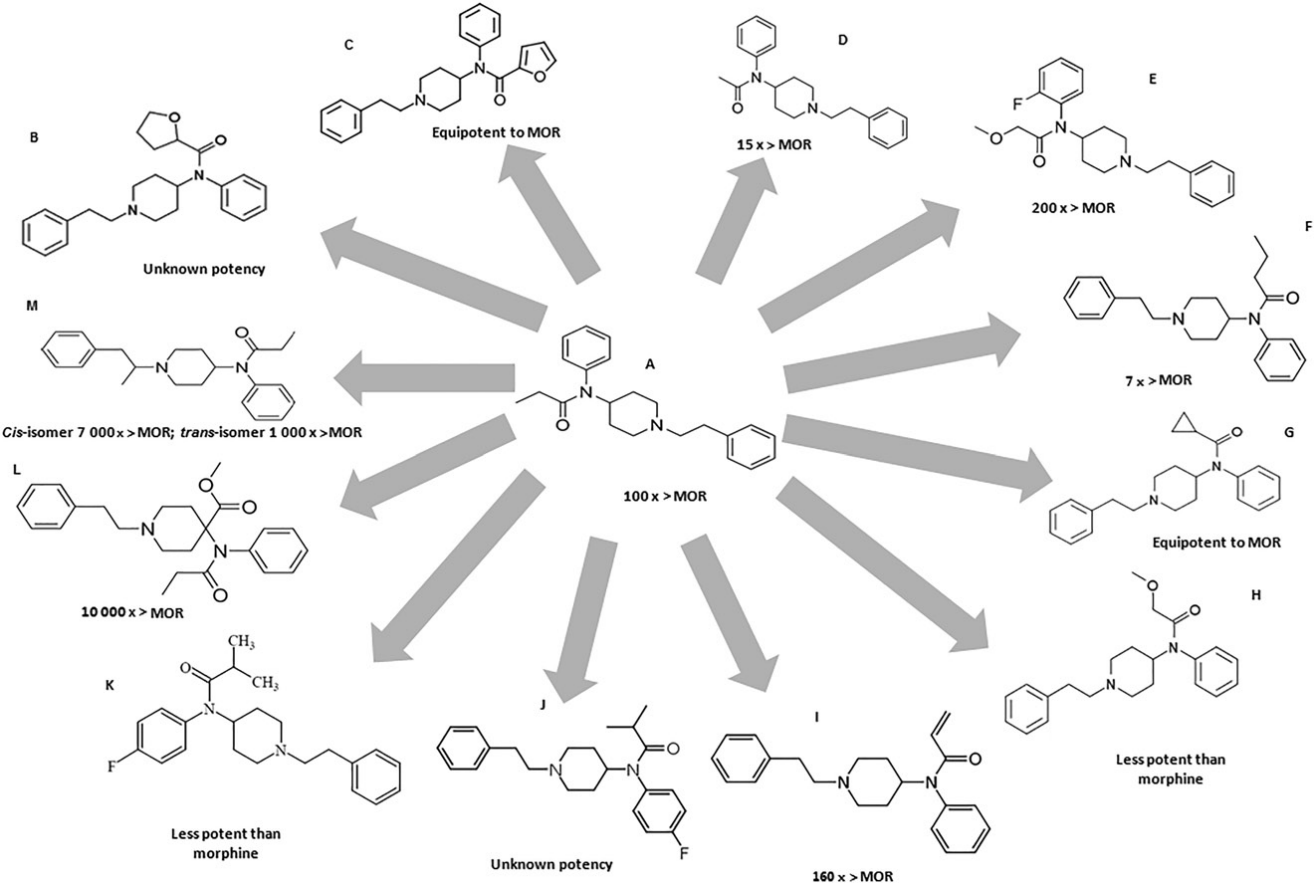
Chemical structure of fenatanyl and their analogues: (A) fentanyl; (B) tetrahydrofuranylfentanyl (THFF); (C) furanylfentanyl; (D) acetylfentanyl; (E) ocfentanil; (F) butyrfentanyl; (G) cyclopropylfentanyl; (H) methoxyacetylfentanyl; (I) acrylfentanyl; (J) *para*-fluoroisobutyrfentanyl; (K) *para*-fluoroisobutyrylfentanyl; (L) carfentanil; (M) α-methylfentanyl. The compounds’ potency has been compared to that of morphine (MOR).

Fentanyl (Figure 1A) was synthesized for the first time in Belgium, December 1960, by Dr. Paul Janssen and the Janssen Company Beerse. Later, some derivates were synthesized (the so-called Fentanyls), such as sufentanil, alfentanil and remifentanil approved for pharmaceutical use in humans, and carfentanil and thiofentanil approved for wild animals [14]. Most of fentanyl illicit analogues are usually manufactured in China and exported to all over the world [15]. Fentanyl was placed under international control as a Schedule I substance in 1964 under the Single Convention on Narcotic Drugs of 1961, and the referred analogues were also included later [16]. Nowadays, fentanyl is considered a Schedule II substance [17]. Due to the high potency of these analogues, overdoses may occur at low doses. This fact unable fentanyl analogues to be detected in routine toxicological analysis [18].

Fentanyl is a high potency opioid, which is widely used as an anaesthetic, sedative and analgesic, with about the 100 times the potency of morphine and 40 times the potency of heroin. Such drugs often produce high dependence among the users and are susceptible to abuse. It was found that the risk of overdose with a fentanyl injection was two times higher than that with heroin, and eight times higher than other prescription opioids [16,19]. Significant analgesia may occur with fentanyl plasma concentrations of 0.2 to 1.2 ng/mL in opioid-naive patients and often at concentrations only slightly higher in some opioid-tolerant patients, or much higher in some patients with more marked tolerance. Fentanyl undergoes metabolization via the human cytochrome P450 isoenzyme system, specifically, CYP3A4. Given this, when fentanyl is co-administered with drugs that affect or are metabolized by this isoenzyme, potential drug interactions may occur [14]. Remifentanil is the only member of the fentanyls which is apparently ∼95% metabolized in the blood and tissues by non-CYP enzymes [20].

Common doses of fentanyl are 25–50 µg/h with transdermal patches and 25–50 µg with intranasal administration [21]. Illicit fentanyl and its analogues are produced in the so-called “pill mills”, where there are not submitted to any quality control and are subject of calculation and measurement errors. As expected, these pills can be fatal, particularly with new fentanyl analogues, whose properties or potency are not well known. The toxic effects of fentanyl can become evident with its misuse, as in the increase of the administered dose, or the use of a different route of administration (e.g. extracting the drug from a transdermal patch into liquid to prepare an injection or nasal spray, inhaling volatilized fentanyl or placing a transdermal patch on oral mucous membranes) [16]. Fentanyl and its analogues have been sold in Europe as ready-to-use nasal sprays and e-liquids for vaping, making its use easier and more socially acceptable [22]. Fentanyl analogues are usually obtained by modification or replacement of fentanyl's propionyl chain (acetylfentanyl, acrylfentanyl, butyrylfentanyl, isobutyrylfentanyl, furanylfentanyl, ocfentanil) or replacement of the ethylphenyl moiety (isofentanyl, β-hydroxythiofentanyl) [23].

Tetrahydrofuranylfentanyl (THFF), also known as tetrahydrofuran fentanyl or *N*-phenyl-*N*-[1-(2-phenylethyl) piperidin-4-yl] tetrahydrofuran-2-carboxamide, is a fentanyl derivate and belongs to 4-anilidopiperidine class, as do fentanyl and its analogues [24], and it is also an agonist of the µ-opioid receptors [25]. THFF (Figure 1B) has a chemical structure very similar to furanylfentanyl (Figure 1C), but the furan-ring is saturated, in the case of THFF [24]. This NSO is one of the fentanyl analogues reported to the UNODC Early Warning Advisory (EWA) between 2012 and 2016 in Europe [16]. Information related to this fentanyl analogue seems to be confined to reports from Sweden and there was a total of 14 reports of death related to THFF between 2016 and 2017. Reduced consciousness, respiratory depression and miosis were some of the symptoms associated with fatalities. It has been seized as a liquid, in powder form and as disk-shaped tablets [16,19]. When overdose occurred, signs were consistent with an opioid overdose as the individuals showed pulmonary congestion and oedema, as well as mild cerebral oedema [24]. Seven metabolites of THFF have been identified, namely, 4-ANPP (a known precursor and intermediate in the synthesis of fentanyl and its analogues), OH-4-ANPP (which resulted from the hydroxylation of the latter), THF-norfentanyl (THFF suffered *N*-dealkylation), hydroxylation of THFF resulted in two other metabolites and two minor metabolites were identified for THFF (one resulting from di-hydroxylation and the other from internal hydrolyses). THF-norfentanyl was found to be a unique biomarker for the ingestion of THFF [24]. According to EMCDDA, this substance was removed from the market in June 2017 [26].

Furanylfentanyl (*N*-(1-(2-phenylethyl)-4-piperidinyl)-*N*-phenylfuran-2-carboxamide) (Figure 1C) was synthesized and patented in 1986, but only made its appearance in illicit market in 2015 [27]. This NSO was reported in Asia, Europe and North America [16]. The common doses for oral administration include 0.5–0.9 mg and for insufflation include 0.4–0.8 mg [21]. Although, most fentanyl analogues are mainly metabolized by *N*-dealkylation, the major metabolite of furanylfentanyl, undergoes amide hydrolyses to produce an intact phenethylpiperidine moiety. This particularity is due to furanylfentanyl's structure [28,29], specifically the aromatic heterocyclic furan that undergoes characteristic bioactivation reactions (such as epoxidation and ring scission). Furanylfentanyl has 14 metabolites, which were obtained through *N*-dealkylation, hydroxylation, amide hydrolysis followed by hydroxylation with glucuronidation or sulfation, dihydrodiol formation followed by hydroxylation or *N*-dealkylation, oxidative *N*-dealkylation and reduction of the keto group, and furanyl ring opening and carboxylation [23]. This compound has a potency similar to that of fentanyl [30]. Reports of intoxication with furanylfentanyl usually show brain oedema and pulmonary oedema, in the autopsy. Survivors to fentanyl intoxication, received treatment promptly, what suggests that, if a person is treated immediately, a fatal outcome might be avoided [31]. This substance was removed from the market in January 2017 according to EMCDDA [26].

Acetylfentanyl (*N*-(1-phenethylpiperidin-4-yl)-*N*-phenylacetamide) (Figure 1D) is an NSO, a fentanyl analogue with a substitution of the *N*-propionyl moiety for an acetyl moiety [27]. It was first reported to the UNODC Early Warning Advisory in 2013, in Asia, ENA and was placed under international control in 2016 [16]. This fentanyl analogue has some similarities with heroin, namely, colour, consistency and pharmacologic activity. It shows greater activity on µ-receptors than morphine (about 15 times) and heroin (5 times) and three times lower than fentanyl and other analogues [24,25]. This compound has been sold as powder form and declared as acrylic paint phenolic resin, as counterfeit Xanax® tablets, mislabelled pills and “bath salts” [16]. Other reports include the use of propylene glycol electronic cigarettes filled with acetylfentayl, labelled as “synthetic opium”, as well as its mixture with alcoholic beverages. “China town” and “Synthetic heroin” are some of the street names used to referrer to acetylfentanyl [32,33]. It is often administered intravenously and seems to be more liable to cause death when consumed through this route of administration [34]. Common oral doses for oral administration of acetylfentanyl include 3–5 mg [21]. Symptoms description from case reports include weak response, altered mental status, followed by respiratory depression, pinpoint pupils, hypoxemia and a Glasgow Coma Scale score of 6. This medical condition was further diagnosed with opioid toxidrome. It seems to have the same pharmacologic effects of other opioids, like analgesia, euphoria, miosis and potentially fatal respiratory depression [32,33]. Acetylfentanyl has 32 metabolites obtained through *N*-dealkylation, followed by hydroxylation, monohydroxylation preferably at the ethyl linker, followed by glucuronidation or sulfation, dihydroxylation followed by glucuronidation or sulfation, monohydroxylation and carbonylation, dihydrodiol formation, dihydroxylation with methylation at the phenyl ring followed by glucuronidation or sulfation, as well as amide hydrolysis followed by hydroxylation. The major metabolite seems to be nor-acetylfentanyl, which is generated by *N*-dealkylation at the piperidine nitrogen resulting in a loss of the phenethyl moiety [23]. According to EMCDDA, this substance was removed from the market in December 2015 [26].

Ocfentanil (*N*-(2-fluorophenyl)-2-methoxy-*N*-[1-(2-phenylethyl)piperidin-4-yl]acetamide), also known as A-3217 (Figure 1E), is a fentanyl analogue reported to the UNODC EWA in 2013, in Europe [16]. It has a similar structure to fentanyl with the addition of a methoxy group instead of a methyl group on the acetamide function and a fluorine atom on the benzene ring [35]. This analogue has dose-dependent analgesic and respiratory depression effects and 3 µg/kg seems to produce the same level of analgesia as 5 µg/kg of fentanyl, in humans [35]. Ocfentanil was found to be 2.5 times as potent as fentanyl an analgesic and around 200 times as potent as morphine, concerning its analgesic properties [36]. Users refer that ocfentanil is less able to produce euphoria when compared to other opioids, which is a factor of discontent [37]. Reports of this analogue include the following routes of administration: sniffing, smoking and intravenous injection [27,29]. Concerning biotransformation, ocfentanil undergoes *O*‐demethylation followed by hydroxylation, *O*‐demethylation followed by glucuronidation, hydroxylation and *O*‐demethylation. The major metabolite of ocfentanil seems to be *O*-desmethyl ocfentanil [35].

Butyrfentanyl (*N*-[1-(2-phenethyl)-4-piperidinyl]-*N*-phenylbutramide) (Figure 1F) is a potent short-acting fentanyl analogue which was reported to the UNODC EWA in 2015, in Asia, Europe and North America [16]. Its chemical structure differs from fentanyl only by one methyl group [38]. A report of overdose with butyrfentanyl describes symptomatology like haemoptysis, acute lung injury (ALI), hypoxic respiratory failure and diffuse alveolar haemorrhage. Pulmonary oedema and ALI are commonly described in opioid overdose, but diffuse alveolar haemorrhage is far less common [38]. When combined with acetylfentanyl, even with relative low doses of the latter, the intoxication resulted in death, probably due to the double action of central nervous system (CNS) depression, which increases the liability of life-threatening hypoventilation and/or fatal respiratory depression following abuse [39]. Studies on animals provide evidence that suggests that butyrfentanyl is seven times more potent than morphine but only 0.13 the potency of fentanyl. It can be snorted and has been sold as what users believed to be acetylfentanyl [38,40]. Concerning its metabolism, carboxy and hydroxybutyrfentanyl were identified as the most abundant metabolites. Butyrfentanyl seems to undergo postmortem redistribution and concentrations in forensic death cases should be interpreted with this in mind [40].

Cyclopropylfentanyl (*N*-phenyl-*N*-[1-(2-phenylethyl)piperidin-4-yl] cyclopropanecarboxamide) (Figure 1G) differs from fentanyl by replacement of the propionamide group of fentanyl with a cyclopropanecarboxamide group. This NSO is also structurally related to butyrfentanyl [41]. Street names of cycloprpylfentanyl include “cyclopropyl” (Belgium), “synthetic heroin” (Belgium), “4-me-MAF” (Sweden), and “MAF” (Poland). It has been detected in powders and, to a lesser extent, in liquids and in tablets. An amount of 1.6 kg of powder containing cyclopropylfentanyl was seized in 26 cases that were reported by Latvia (18), Poland (2), Sweden (4) and the UK (2). The seized powders were reported to be white or off-white in colour [41]. This NSO is expected to have the opioid-like effects of SNS depression. The risk of severe respiratory depression may be greater due to difficulty in diluting the substance, lack of experience with its effects and dosing, the use of other CNS depressants at the same time (such as other opioids, benzodiazepines, gabapentanoids and alcohol), lack of tolerance to opioids and using the substance alone (such as at home) which would make it more difficult for individuals to call for help in the case of overdosing. It is important to remark that cyclopropylfentanyl does not have a recognized human or animal medical use [41]. Drug Enforcement Administration temporary placed cyclopropylfentanyl in Schedule I, to be effective from 4 January 2018, until 4 January 2020 [17]. According to EMCDDA, this substance was removed from the market in December 2017 [26] and its potency is similar to that of morphine.

Methoxyacetylfentanyl (2-methoxy-*N*-phenyl-*N*-[1-(2-phenylethyl) piperidin-4-yl]acetamide) (Figure 1H) differs from fentanyl due to the replacement of the propionamide group with a 2-methoxyacetamide group. It is also structurally related to ocfentanil [42]. This NSO (less potent than morphine) was reported to the UNODC EWA between 2012 and 2016 in Europe [16]. Street names for methoxyacetylfentanyl include “MAF” (Belgium), “methoxy” (Belgium), and “synthetic heroin” (Belgium). It has been identified in the form of powders and liquids, and, to a lesser extent, tablets [42]. According to the EMCDDA, 33 seizure cases were reported by seven Member States: Belgium (1), Denmark (1), Hungary (1), Latvia (7), Sweden (20), Slovenia (1), the UK (1) and Norway (1) [42]. Like other opioid analgesics, the most serious acute health risk related to methoxyacetylfentanyl is expected to be respiratory depression, which in overdose could lead to apnea, respiratory arrest and death. This NSO does not have a recognized human or animal medical use [42]. According to EMCDDA, this substance was removed from the market in December 2017 [26].

Acrylfentanyl (*N*-phenyl-*N*-[1-(2-phenethyl)piperidin-4-yl]prop-2-enamide) (Figure 1I), also known as acryloylfentanyl, is a fentanyl analogue reported to the UNODC EWA in 2016, in Asia and Europe [16,43]. Regarding its analgesic properties, it was reported that acrylfentanyl is 160 times more potent than morphine and has greater affinity for the µ-receptor than fentanyl. This analogue seems have a greater ability to induce long lasting analgesia when compared to fentanyl or morphine [44]. Acrylfentanyl has been administrated in the form of nasal spray, containing only acrylfentanyl or mixed with other drugs; in the form of tablets, which can be crushed and snorted, besides the conventional form of administration; by intravenous injection; and in the form of capsules [44]. Common doses for insufflation with acrylfentanyl range from 12.5 to 25 µg [21]. Intoxication with acrylfentanyl has resulted in the classic opioid intoxication symptoms (respiratory depression, breathing arrest, partial or complete loss of consciousness and miosis). This analogue seems to have the particularity to exert on first time users, rather than on regular users only, like the other fentanyl analogues [44].

Acrylfentanyl's metabolism has been studied, suggesting that it undergoes *N*-dealkylation at the piperidine nitrogen producing a major nor-metabolite. Monohydroxylated metabolites were either hydroxylated at the ethyl linker, the *N*-phenyl ring or acryl moiety or the piperidine ring. One of the two dihydroxy metabolites is dihydroxylated at the *N*-phenyl ring or the acryl moiety, while the other one is hydroxylated once each at the ethyl liker and the adjacent phenyl group. Two dihydrodiol metabolites were detected, one carrying the two hydroxy groups at the ethylphenyl ring, while the other one on the *N*-phenyl ring. Both dihydroxylated/methylated metabolites carried the hydroxy and methoxy group at the phenyl ring of the phenethyl moiety. A desacrylated metabolite generated by amide hydrolysis was also identified and is characterized by the intact phenethylpiperidine moiety. Three glucuronic metabolites were also identified in non-hydrolysed urine samples [23].

*para*-Fluoroisobutyrfentanyl (*N*-(4-fluorophenyl)-*N*-[1-(2-phenylethyl)piperidin-4-yl]butanamide; 4-FBF or p-FIBF) (Figure 1J) is a fentanyl analogue that was reported to the UNODC EWA in 2016 in North America [16]. Regarding its metabolism, a study described 17 metabolites generated by *N*-dealkylation, hydroxylation (six metabolites), followed by glucuronidation, dihydroxylation, dihydrodiol formation, dihydroxylation with methylation (two metabolites) followed by glucuronidation, amide hydrolysis, oxidative *N*-dealkylation, and further reduction of the keto group, carboxylation and carbonylation. Nine metabolites were observed in the hepatocytes, of which the nor-metabolite was the major metabolite in the 5 h sample, followed by the monohydroxylated metabolites. In hydrolysed urine, 11 metabolites were detected and the only difference from the hepatocytes was that that hydroxymethoxy 4-fluoro-isobutyrylfentanyl was also abundant. In non-hydrolysed urine, two additional glucuronides were detected, being completely cleaved by hydrolysis [23].

*para*-Fluoroisobutyrylfentanyl or (*N*-(4-fluorophenyl)-2-methyl *N*-[1-(2-phenylethyl)piperidin-4-yl]propanamide) (Figure 1K) was notified on 26 August 2016 by the EMCDDA. This fentanyl analogue (less potent than morphine) has been associated with 16 deaths reported by one-member state. This compound was often associated with other drugs, but it was found to cause or have contributed to at least 13 deaths. This opioid has no recognized human or veterinary medical use [45]. Like other NSO, this fentanyl is often sold online as a “research chemical” or as a “legal” replacement to illicit opioids, as powder, liquid (e.g. in ready-to-use nasal spray) and blotter. *para*-Fluoroisobutyrylfentanyl was also sold under the guise of heroin or in mixtures with other opioids like heroin and furanylfentanyl [45].

Carfentanil (methyl 1-(2-phenylethyl)-4-(*N*-propanoylanilino)piperidine-4-carboxylate) (Figure 1L) was synthesized for the first time in 1974. This synthetic opioid is considered the most potent opioid commercially available in the world and it is not under international control. Carfentanil is approved for veterinary use in large animals and is estimated to be about 10 000 times more potent than morphine, 4 000 times that of heroin and 100 times that of fentanyl [16,21]. It is the active ingredient of *Wildnil* [15]. This NSO has been increasingly sold mixed with or under the guise of heroin [21] and has been identified in mixtures with cocaine, heroin, fentanyl, furanylfentanyl, acrylfentanyl, caffeine and antihistamines, as well as laced with ketamine [15]. Since the therapeutic index of carfentanil is higher than that for morphine and fentanyl, the uncontrolled dosing is probably the reason of the massive overdosing. This fentanyl analogue has already been weaponized in the past (October 2002) and used to control a hostage situation in a Moscow Theater. Besides incapacitating everyone in the room, it also resulted in the death of 15% of the hostages, due to unavailability of naloxone for several hours [46]. The metabolism of carfentanil has been studied on human hepatocytes. According to this study, carfentanil seems to undergo, as expected, *N*-dealkylation (three metabolites), monohydroxilation (three metabolites), *N*-oxidation (two metabolites) and a combination of *N*-oxidation and hydroxylation (one metabolite). Other metabolites result from carbonylation, or ketone formation, ester hydrolysis and glucuronidation. In total, 12 metabolites of carfentanil were identified [20]. According to EMCDDA, this substance was removed from the market in July 2017 [26].

α-Methylfentanyl (Figure 1M) is a fentanyl analogue which was placed under control by the 1961 Single Convention on Narcotic Drugs in 1988 [47]. This analogue, like other fentanyls, has been identified in heroin formulations as an adulterant, causing sudden deaths. Animal studies of α-methylfentanyl showed that this analogue has a narrow therapeutic index than fentanyl [48]. However, it is believed to be significantly more potent than fentanyl [18]. The *cis-*isomer is 7 000 times more potent than morphine and 7 times more potent than *trans*-isomer [49]. In the past, this fentanyl analogue has been associated with several overdose deaths in southern California from respiratory paralysis [18]. Also, it has been identified in a formulation mention as “China White” and believed to be responsible for several deaths [50]. The use of this drug was popularized and spread in Russia since 1991, where a group of chemistry students discovered a simplified synthetic route that used phosgene instead of phenethylamine [47]. As indicated above, the emerging NSO pose an alarming threat to public health. There are still fentanyl analogues which are not placed under control, as evidenced by Table 1.

**Table 1.** Fentanyl analogues reported to the United Nations Office on Drugs and Crime (UNODC) Early Warning Advisory (EWA), not yet placed under control and fentanyl and analogues controlled under the 1961 Single Convention on Narcotic Drugs [47].

Control status	Fentanyl analogues
Reported to the UNODC EWA—not yet placed under control	Acrylfentanyl
	*para*-Fluoroisobutyrfentanyl
	Butyrfentanyl
	Furanylfentanyl
	Ocfentanil
	Carfentanil
Controlled under the 1961 Single Convention on Narcotic Drugs	Remifentanil
	Acetylfentanyl
	α-Methyl-thiofentanyl
	β-Hydroxyfentanyl
	β-Hydroxy-3-methylfentanyl
	3-Methylthiofentanyl
	*para*-Fluorofentanyl
	Thiofentanyl
	Acetyl-α-methylfentanyl
	α-Methylfentanyl
	3-Methylfentanyl
	Alfentanil
	Sufentanil
	Fentanyl

**Figure 2.**
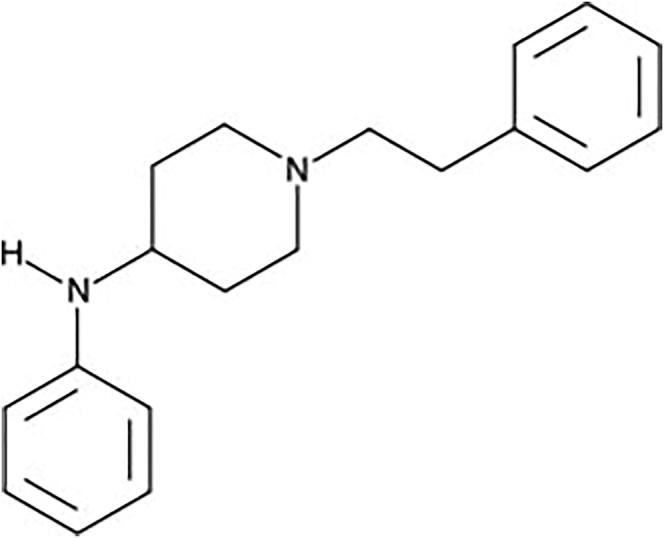
4-ANPP.

4-ANPP (4-anilino-*N*-phenethyl-piperidine) (Figure 2), or despropionylfentanyl, is a known precursor and a metabolite of all NSO fentanyl related. This metabolite has been detected as a fentanyl metabolite in plasma of patients given fentanyl, but it has not been reported in urine [28]. Expectations for new fentanyl analogues that might emerge are the normetabolite, one or several hydroxy metabolites and/or a hydroxymethoxy metabolite will be prevalent. Despite this, some analogues can show a significantly different metabolism, therefore, it is important to confirm the predictions with comprehensive metabolite identification studies [23].

### Other synthetic opioids

**Figure 3.**
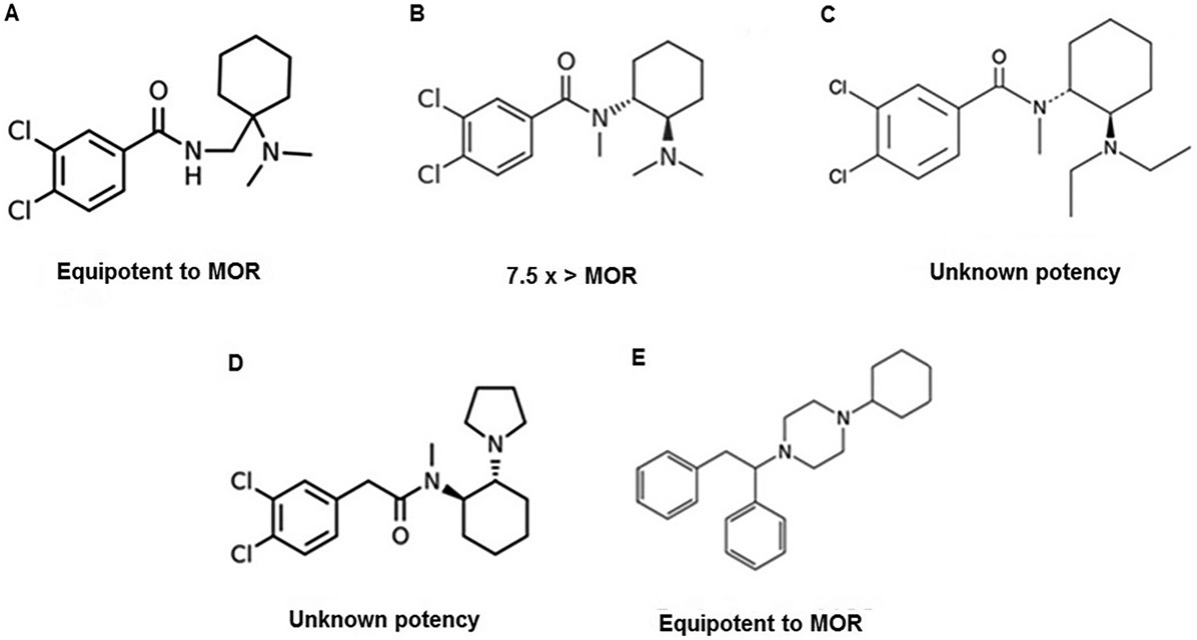
Other novel synthetic opioids: (A) AH-7921; (B) U-47700; (C) U-49900; (D) U-50488; (E) MT-45. The compounds’ potency has been compared to that of morphine (MOR).

Besides fentanyl and its analogues, other synthetics opioids, such as U-47700, appeared in the market. AH-7921 (3,4-dichloro-*N*-(1-(dimethylamino)cyclohexylmethyl) benzamide) (Figure 3A) is an analgesic agonist of the µ-opioid receptors, with some action on k-opioid receptors, and was patented by Allen and Hanburys Ltds. in the mid-1970s. The name “doxylam” was proposed for this analgesic but it was never sold commercially. Also, this synthetic opioid has no industrial use. Besides “doxylam”, names like “doxylan” and “CN 2924 29 98” (CAS - Chemical Abstracts Service - Number) are used to refer to AH-7921 [51–53]. The hydrochloride salt of AH-7921 is a white solid and the free amine of AH-7921 is also reported to be a solid [54]. AH-7921 was first identified in July 2012 in the UK and was formally notified to the EWA in August 2012. Following an assessment of the available information on AH-7921, the EMCDDA and Europol submitted a Joint Report on AH-7921 to the Council of the European Union, the European Commission and the European Medicines Agency [54,55]. The minimal dose of AH-7921 required to suppress pain seems to be 1.25 ± 0.8 mg/kg, which is approximately the same as for morphine, and lower than that for codeine [53]. Animal studies have concluded that AH-7921 has an activity comparable to morphine regarding analgesia, hypothermia, addictive behaviour and respiratory depression properties [51,52]. AH-7921 has half of the safety margin of morphine, suggesting that it has a greater ability to cause adverse effects. This NSO has a structure similar to fentanyl and phencyclidine, therefore being a potent analgesic [51]. As unwanted effects reported by users are nausea and vertigo induced by movement (that could be minimized by having a meal 2–4 h prior to ingestion), “opiate glow”, alertness, occasional itching, nausea and tremors after sublingual administration and re-dosing of a solution of powder AH-7921 in lemon juice and warm water. Experience with AH-7921 was described as predictable and repetitive. Withdrawal symptoms have been described as feelings of depression and mild insomnia and have been classified to be worse than those for morphine [51]. Results from a study in mice indicate that AH-7921 interacts *in vivo* with brain-penetrating serotonergic and adrenergic drugs. The antinociceptive effects of this synthetic opioid seems to be prolonged when co-administrated with intracerebroventricular serotonin, while noradrenaline seems to attenuate the antinociceptive effects of AH-7921, and the same for morphine [56]. AH-7921 is often used as substitute for heroin, and also combined with synthetic cannabinoids and α-pyrrolidinobutiophenone in an illegal herbal-type drug sold over the internet in Japan [52,53]. Seizures of this compound usually report the form of powder [51,54]. Regarding its metabolism, AH-7921 seems to undergo demethylation, less pronounced hydroxylation and combinations of different biotransformation [57]. Possible forms of administration are nasal insufflation, sublingual application, intravenous injection, a combination of insufflation and oral consumption or rectal administration (in the form of powder, tablet or capsule) and the available doses range from 10 to 150 mg [51].

There are some concerns about the similarity of AH-7921 street name “doxylam” with “doxylamine” which refers to an antihistaminic with sedative properties. The consumption AH-7921 instead of doxylamine could lead to an unintentional overdose [51,54]. According to EMCDDA, this substance was removed from the market in December 2013 [26].

U-47700 (3,4-dichloro-*N*-[2-(dimethylamino)cyclohexyl]*N*-methylbenzamide) (Figure 3B) is a non-fentanyl based synthetic opioid developed by the Upjohn Company in the 1970s and it is a structural isomer of the opioid analgesic AH-7921 (3,4-dichloro-*N*{[1-(dimethylamino) cyclohexyl]methyl}benzamide) [58,59]. This NSO binds to the µ-opioid receptor with high affinity, much less well to the κ-opioid receptor and poorly to the δ-opioid receptor. The opioid morphine has a much lower affinity to the three receptors. U-47700 is reported to be the compound most selective for the µ-opioid receptor among all studied [60]. This synthetic opioid seems to have opiate-like adverse effects including pinpoint pupils, respiratory depression, cyanosis and depressed consciousness [59,61]. U-47700 is thought to be approximately 7.5 times more potent than morphine [58,59,61]. This NSO has been on the online market, promoted as heroin or as an oxycodone substitute, as itself, or in combination with other drugs such as fentanyl, under the street names “U-47700”, “Fake morphine”, “U4”, “pink” and sometimes referred to as “synthetic cocaine” [61,62]. This drug is usually sold as a powder or as liquid to use in inhalers. Users report the use of administrations routes like oral, insufflation, intravenous, rectal and via an inhaler using a liquid solution [61]. Common doses of U-47700 use range from 7.5 to 15 mg [21,62]. This opioid analogue has a duration of action of 5 to 7 h when taken orally, 3 to 4 h when snorted and 1 to 2 h when administered intravenously [62].

Regarding its metabolism, the demethylated metabolite was found to be the most abundant, followed by the bisdesmethyl, desmethyl hydroxyl and bisdesmethyl hydroxy forms [61]. Postmortem findings of intoxication with U-47700 include pulmonary oedema, cardiomegaly and cerebral oedema [63]. The analytical identification has been performed by targeted liquid chromatography–mass spectrometry (LC-MS) and untargeted accurate-mass quadrupole time-of-flight (QTOF)-LC-MS, and the results were initially misinterpreted as AH-7921, because of similarities in transition ions and retention times. High-performance liquid chromatography coupled with diode-array detection (HPLC-DAD), on the other hand, showed different retention time and UV spectra. Analysis by triple quadrupole/linear ion trap LC-MS showed that U-47700 and its precursor AH-7921 share some primary product ions, therefore, when relaying on a targeted ion transition method, specific transitions *m/z* 329 > 81 and *m/z* 329 > 204 are recommended for accurate identification of U-47700. Analysis through high accuracy QTOF-MS was able to determine the empirical formulae of *N*-desmethyl and *N*,*N*-didesmethyl metabolites [64].

U-49900 (3,4-dichloro-*N*(2-(diethylamino)cyclohexyl)-*N*-methylbenzamide) (Figure 3C) is the diethyl analogue of U-47700, also developed by Upjohn [60]. Two months after the Drug Enforcement Administration (DEA) has placed U-47700 into Schedule I of the Controlled Substances Act, U-49900 was identified through online drug forums and research chemical vendor websites [65]. Other NSO are scheduled by DEA, mostly as level I, as evidenced by Table 2.

**Table 2.** Novel synthetic opioids schedule by Drug Enforcement Administration in 2018 [66].

Substance	Schedule^a^
3-Methylfentanyl	I
3-Methylthiofentanyl	I
Acetyl fentanyl	I
Acetyl-α-methylfentanyl	I
Acetyldihydrocodeine	I
AH-7921	I
α-Methylfentanyl	I
α-Methylthiofentany	I
Acrylfentanyl	I
β-Hydroxy-3-methylfentanyl	I
β-Hydroxyfentanyl	I
β-Hydroxythiofentanyl	I
Butyryl Fentanyl	I
Carfentanil	II
Cyclopropylfentnyl	I
Fentanyl	II
Furanyl fentanyl	I
Isobutyryl fentanyl	I
Methoxyacetylfentanyl	I
Ocfentanil	I
*para*-Fluorofentanyl	I
U-47700	I
Valeryl fentanyl	I

a Schedule I means the drug has a high potential for abuse, has no currently accepted medical use in treatment in the US or lacks accepted safety for use of the drug or other substance under medical supervision; Schedule II means the drug has a high potential for abuse, has a currently accepted medical use in treatment in the US or a currently accepted medical use with severe restrictions, or abuse of the drug may lead to severe psychological or physical dependence.

There are very few references for this synthetic opioid. Some adverse effects of this NSO were reported, like loss of smell, loss of taste, nerve damage specially on the left side of the body and the appearance of a “foam-like substance” in the lungs that ended up being excreted by cough [66]. The U-49900 was not considered a good substitute for U-47700, since the users concluded that it is inert in terms of euphoria and analgesia, even at high doses. Doses of U-49900 required for bioaction are reported to be much higher than those for U-47700 [60]. Individuals who described their experience on the referred forum, attributed little or no effect to U-49900 at doses ranging from 5 to 75 mg by intravenous injection, insufflation or oral ingestion, and consider its odour as caustic [66]. A study regarding its metabolism concluded that *N*-Desethyl-U-49900 was the primary metabolite of U-49900 following microsomal incubations, while *N*,*N*-didesethyl-*N*-desmethyl-U-49900 was the most abundant in a urine specimen. U-47700 and U-49900 seem to undergo similar metabolic pathways, resulting in common metabolite and isomeric species: *N*,*N*-didesmethyl-U-47700 and *N*,*N*-didesethyl-U-49900 (similar in formula and structure and result in a common metabolite 3,4-dichloro-*N*-(2-aminocyclohexyl)-*N*-methylbenzamide). This consideration should be taken into account in cases involving these two analogues [65].

U-50488 or (2-(3,4-dichlorophenyl)-*N*-methyl-*N*-[(1R,2R)-2-pyrrolidin-1-ylcyclohexyl] acetamide) (Figure 3D) is an NSO developed by Upjohn in the 1970s as a κ-selective derivative of U-47700 [67]. Studies in animals have shown that U-50488 causes diuresis and dysphoria rather than respiratory depression or constipation [67]. It shows agonist activity on the κ-opioid receptor, with some reported μ-opioid receptor respiratory antagonist effects [30]. Although the toxicological profile and toxicoepidemiology of U-50488 are unknown, the structural similarity of U-50488 to U-47700 poses a risk [67].

MT-45 (1-cyclohexyl-4-(1,2-diphenylethyl)piperazine), also known as IC-6, is an *N*,*N*-disubstituted 4-(1,2-diphenylethyl) piperazine (Figure 3E), and shows a different chemical structure from other opioid agonists [6,52]. This synthetic opioid was developed in the 1970s by the Dainippon Pharmaceutical Co. in Japan [6]. Like other NPS, MT-45 is often sold online as a “research chemical” and has been combined with synthetic cannabinoids, cathinones and a phenethylamine derivate in chemical and herbal products [6,52]. It is usually sold in its dihydrochloride salt form [6]. Regarding its analgesic properties, it seems to be comparable to morphine [52]. S(+) enantiomer and racemate MT-45 were found to be more potent than morphine. Also, the S(+) isomer seems to be more potent than the racemate and highly more potent than R(–) isomer. Regarding its structure-activity, the nitrogen at 4-position seems to play a key role in determining the morphine-like effect of MT-45 [68]. This NSO has a complex and not totally understood action, since it affects the opioid and other non-opioid receptors [6].

Possible administration routes include oral, insufflation, intravenous and intramuscular and intrarectal. MT-45 has been reported as a white powder. Common dosages of oral administration usually range from approximately 50 mg for opioid naive users up to 250 mg for highly tolerant individuals [6]. Adverse effects reported by users include CNS depression typical effects, nausea, itching, bilateral hearing loss, possible withdrawal symptoms and dissociative-like symptoms [6]. Case reports regarding the use of MT-45 described unexpected reactions like dry and scaly skin, angular cheilitis, cracks on the fingers and under the feet, redness and moist maceration of the groins and armpits, and total alopecia, as well as loss of taste, smell and chills almost constantly, hair depigmentation, transverse white Mees' lines on the fingernails, eyebrows and eyelashes turning completely white, elevated levels of the enzymes aspartate transaminase (AST) and alanine transaminase (ALT), sudden hearing loss and deafness, irritated and dry eyes, culminating in loss of vision and almost blindness, imposing the need for cataract surgery performed on both eyes [69]. MT-45 has also been associated with cases that resulted in deeply unconsciousness, apnea, decreased respiratory rate, cyanosis, neurological disturbances such as paraesthesia in hands and feet, difficulties to grip and coordinate hand movements, balance disturbances and vision impairment [70]. MT-45's metabolism has been studied, using rat hepatocytes and LC systems coupled with high resolution mass spectrometry (LC–HRMS). Phase I and II metabolites were identified, products of monohydroxylation, dihydroxylation and *N*-dealkylation, as well as, glucuronide conjugation of monohydroxylated and dihydroxylated metabolites. Hydroxylated MT-45 has demonstrated to be bioactive, suggesting it may contribute to the overall pharmaco-toxicological profile of MT-45 reported [71]. According to EMCDDA, this substance was removed from the market in June 2014 [26].

W-18 (4-chloro-*N*-[1-[2-(4-nitrophenyl)ethyl]-2-piperidinylidene]-benzenesulfonamide) and W-15 (4-chloro-*N*-[1-(2-phenylethyl)-2-piperidinylidene]-benzenesulfonamide) previously thought to be analogues of the opioid fentanyl, have some differences in key respects chemically from fentanyl. The presence of an aryl sulfonamide group, instead of the tertiary amine, renders the piperidine nitrogen atom nonbasic [72]. This substance was first reported to the EWA on 10 September 2014 in Sweden [73]. W-18 and W-15 show no significant activity in opioid receptors, even at high concentrations. For this reason, they are not considered a synthetic opioid. As a matter of fact, they seem to have some affinity to non-opioid receptors, such as 5-HT2A, 5-HT2B, 5-HT2C and 5-HT6 serotonin receptors, mostly as antagonists; benzodiazepine receptors (BZP and PBR); and other miscellaneous targets. When administrated to mice, they did not show the classical opioid behaviour (hyperlocomotion or Straub tail) and naloxone failed to reverse the burrowing behaviour observed [72]. W-18 seems to be extensively metabolized, which results in multiple monohydroxylated and dihydroxylated metabolites as well as a dealkylated and an amino metabolite from reduction of the nitro group [72].

### Toxicological aspects

The emergence of very potent synthetic opioids, which present serious risks to public health, is one of the elements highlighted in the annual report of the EMCDDA about the update from the EU Early Warning System [74]. More than 700 people per month died from an overdose in 2015 in the EU, Norway and Turkey, representing an increase of 6% over 2014. During 2016–2017, there was a large increase in the availability of NSO in parts of Europe. Most of these substances come from the highly potent fentanyl family and are of special concern to public health because they pose a high risk of life-threatening poisoning, as an overdose can quickly stop a person from breathing. This makes them especially dangerous to users, particularly as many will be unaware that they might be sold as heroin and other illicit opioids or even sold as falsified (fake) medicines. Here, we provide an overview of the current situation with new fentanyls in Europe, which in 2016–2017 were involved in more than 250 deaths, as well as reviewing the key findings of the risk assessments conducted by the EMCDDA on five of them during 2017. The recent rise in drug-related deaths, the changes in the market for new psychoactive substances and the problems in marginalized communities, as well as the recent developments in the new synthetic opioids require a greater attention and co-operation from all authorities. However, the biggest boom in the consumption of new synthetic opioids is happening in the US. Authorities point out that of the 64 000 abuse substances deaths in 2016, 20 145 were for synthetic opioids, a category dominated by fentanyl. In fact due to the high number of occurrences, the concerns of the scientific community have increased, and as such the number of recent publications on this matter as increased as well; this situation has led to improvements in analytical tools, allowing a better understanding of these compounds in what concerns their *in vivo* and *postmortem* effects [75–83]. The following lines summarize a compilation of the case reports found in the PubMed database using all names of the compounds mentioned (Supplementary Table S1), with or without the expression “case report” [18,28,30,34,35,39,40,44,49,52,53,64,84–133]. Due to the complexity of the search, the detection of fentanyl was not considered; only cases where NSO other than fentanyl were detected were subjected to review. This table represents a compilation of the toxic or lethal concentrations of synthetic opioids and some metabolites found in the cases studied. To facilitate their understanding the case reports were organized by compound involved in the intoxication.

Despite the growing popularity of these compounds, for some of them there are still no reports and, in general, most reports have emerged in recent years. However, compounds such as acetylfentanyl, furanylfentanyl and U-47700 are the most representative of this group of substances. Although the analysed samples present a great diversity, highlighting the works of Vorce et al. [53] and Staeheli et al. [40], the most frequent are urine and whole blood samples.

It can be concluded that most cases are from fatal poisonings. On the other hand, it is complicated to define a concentration as being lethal or only toxic because its effect depends on several factors and varies from individual to individual. In fact, in most of these fatal cases other drugs were present besides the opioid. The concentrations found in these cases were below 0.1 ng/mL. The two works of Goggin et al. [28, 134], stand out for being studied of pain management, instead of the remaining articles. The work of Helander et al. [99] describes the real samples as coming from cases of intoxication, not clarifying the state of the patients. The work of Sofalvi et al. [85] includes a case of driving under the influence of drugs. The autoptic findings described in the cited articles are generally similar, such as respiratory problems and central nervous system depression, a topic more fully described by Olaf Drummer's [81] current and excellent review.

### Toxicological identification of NSO

Given the emerging fatalities resulting from the intoxication with NSO, it is important to assess the drug exposure, using techniques that allow the determination of these drugs and their metabolites in biological specimens. As described in the previous section, the most commonly biological matrices used to detect and quantify these drugs of abuse are blood and urine [135]. However, there are also unconventional postmortem matrices such as tissues, vitreous humour or bile. Moreover, newly described methods used to determine these compounds in alternative samples like oral fluid [136] cannot be applied to real samples from individuals with intoxication or NSO users. All analytical determinations, once the samples arrive at the laboratory, begins with the use of screening methodologies. The true extent of the consumption and/or intoxication with NSO is underestimated due to the lack of routine diagnostic tests since the standard immunoassay screening in the clinical setting does not detect synthetic opioids. In terms of initial screening, the immunoassays-based techniques, like enzyme-linked immunosorbent assay (ELISA) or homogeneous immunoassays, are still the mostly used [24,135]. Opioid immunoassays provide a fast outcome using a simple procedure and there is a wide availability of immunoassay platforms which can detect multiple drugs within the same class, due to cross-reaction with the antibody. On the other hand, these immunoassays may fail to detect synthetic opioids due to slight or no cross-reactivity with traditional opioids. For instance, fentanyl and related synthetic compounds are not detected by commercial morphine- or oxycodone-specific opiate immunoassays [135,137]. As a number of fentanyl analogues demonstrate substantial cross-reactivity for the fentanyl antibody on ELISA, it seems to be an effective method to detect synthetic opioids, but it would not be able to distinguish between fentanyl and acetylfentanyl [32,34]. Commercial immunoassays have been studied concerning their ability to detect fentanyl and its analogues, including the Thermo DRI® Fentanyl Enzyme Immunoassay (Thermo Fisher Scientific, Waltham, MA, USA), the ARK™ Fentanyl Assay homogeneous enzyme immunoassay (ARK Diagnostics, Inc., Freemont, CA, USA), and the Immunalysis^®^ Fentanyl Urine SEFRIA™ Drug Screening Kit (Immunalysis Corp., Pomona, CA, USA). This study concluded that the three assays provide a rapid, preliminary screening of a large number of structurally similar designer fentanyls [30]. However, the main problem is the cross-reactivity that is common to the different commercially immunoassays. For example, DRI^®^ Fentanyl Assay was proven to cross-react with acetylfentanyl [138]. However, risperidone and 9-hydroxyrisperidone were also found to cross-react, whereas norfentanyl did not. Fentanyl, acetylfentanyl, risperidone and 9-hydroxyrisperidone all share an intramolecular alkylated piperidine (3-methyl-5-piperidino-2-pentene) that is not present in norfentanyl. This is likely recognized in part by the antibody for the immunoassay. The presence of this moiety could potentially be used to predict cross-reactivity with other fentanyl analogues; however, detection of these compounds has not been analytically evaluated for this assay [29]. Recently, Randox developed a biochip platform that enables the detection of acetylfentanyl, carfentanil, furanylfentanyl, ocfentanil, remifentanil and sufentanil, as well as AH-7921, MT-45 and U-47700 with cut-offs ranging from 0.25 (carfentanil) to 10 ng/mL (U-47700) in urine [81]. However, this assay has not been independently evaluated [29].

Considering the mentioned difficulties, highly specific methods such as gas chromatography (GC) or liquid chromatography (LC) together with mass spectrometry (MS) are used to confirm the exact result of the drug responsible for the positive screening. Obviously, before running chromatographic assays the previous step of sample preparation will undoubtedly be a fundamental step. In fact, this step will be the subject of a thorough revision in this manuscript. LC-MS (or MS in tandem or ion trap) is certainly the most used chromatographic technique for the determination of NSO in detriment of the GC, probably because the latter requires that the molecules are volatile and non-polar (being necessary a derivatization process), which could be a problem, since the compounds are usually metabolized in the liver to more hydrophilic molecules.

Targeted LC-MS/MS methods designed to simultaneously detect fentanyl, fentanyl analogues and other synthetic opioids were first published in 2009 [139,140]. Since then many methods have been developed. An example of them was the recent article published by Salomone et al. [141]. These authors quantify furanyl-fentanyl, 4-ANPP, acetyl-fentanyl, remifentanil, carfentanil, alfentanil, U-47700, fentanyl, sufentanil, and norfentanyl in hair samples (25 mg) by direct injection in LC-MS/MS after previous incubation with 1 mL of methanol at 55 °C for 15 h. These authors archived excellent limits of quantification (1 pg/mg) and recoveries (>71%).

Another example was the one developed by Strayer et al [142]. These authors developed an LC-MS/MS-based method for the multiple detection of 24 fentanyl analogues and metabolites in postmortem blood at sub-ng/mL concentrations, which was successfully implemented at the Montgomery County Coroner's Office/Miami Valley Regional Crime Laboratory in Dayton, Ohio, and demonstrated flexibility and cost- and time-efficiency, as it requires 13.5 min scan time for a single sample and 5–10 min for quantitative and qualitative analysis, with limits of quantification as low as 0.100 ng/mL.

Nonetheless, novel synthetic compounds continue to appear in the market, and therefore, GC-MS and LC-MS/MS procedures present a few drawbacks in the matter, since methods are often targeted and/or are dependent on the availability of mass spectral libraries. In addition, results are usually not obtained in due time in order to allow the directed immediate care of a patient.

LC systems coupled with high resolution mass spectrometry (LC-HRMS) using quadrupole time-of-flight or orbitrap technology can resolve molecular mass to 0.001 atomic mass units, while the conventional MS resolves at 1 atomic mass unit. These techniques allow the identification of compounds lacking mass spectral, by deducing the molecular formula from accurate mass databases [131,143]. Tentative identification of unknowns can be performed without the availability of a reference standard or a library spectrum. Data acquisition is performed in an untargeted fashion, and later on there is the possibility of retrospective analysis to screen for new, and previously undetected, compounds. In recent years, LC-HRMS has been used for the detection of synthetic opioids such as fentanyl analogues (butyrfentanyl, 4-fluorobutyrfentanyl, acetylfentanyl, 4-methoxybutyrfentanyl, furanylfentanyl, acrylfentanyl, 4Cl-iBF, 4F-iBF, THF-F, cyclopentylfentanyl), AH-7921, MT-45 and U-47700 in individual cases, case series, outbreaks and epidemiological surveillance efforts [22,23,28,30,35,50,55,57,68,69,85,87,97–101]. A quite relevant utility is the used of this instrumentation for the elucidation of the metabolic pathways of emerging synthetic opioids, such as AH-7921, butyrfentanyl, carfentanil, U-47700, furanylfentanyl, acetylfentanyl, acrylfentanyl and 4F-iBF [20,23,28,57,128,129,144].

LC-HRMS has clearly been demonstrated to be the most relevant method for analysis of NSO; however, this type of instrumentation is not available in most laboratories yet.

Several factors contribute to the difficulty to detect NPS in screening analyses, such as the limited information about the chemical structure, metabolism and pharmacokinetics of these compounds, the low levels in postmortem blood and urine samples reported with NSO, the lack of cross-reactivity between these new compounds and their metabolites and the existing classes of drugs of abuse, the unavailability of mass spectrum of the compound, which is necessary for MS-based screening assays and the mislabelled illicit drugs (e.g. fentanyl analogues sold as “heroin”), making the clinical histories unreliable and the targeted drug screening less useful. The detection of fentanyl analogues may be difficult by the fact that some compounds share metabolites. Indeed, fentanyl is metabolized into norfentanyl, a metabolite also produced in the case of alfentanil or sufentanil ingestion, which makes forensic distinction virtually impossible in those cases when this metabolite is the only detected compound [20].

As aforementioned, LC-MS/MS holds enormous potentials for improvements, concerning the determination of NSO in biological specimens. Recently, an excellent and comprehensive review about the analytical methods used for this purpose has been made by Marchei et al. [82].

As no reviews on sample preparation techniques for these drugs of abuse have been published so far, and bearing in mind that the greater volume of laboratory work involves sample preparation, we have carried out a thorough and critical review of recently published approaches for the qualitative and quantitative determination of this type of drugs in biological specimens.

### Classic sample pre-treatment techniques applied to determine NSO in biological specimens

Protein precipitation is a widely used technique for pre-treatment of blood [89,104,120,129], but it is also applied in urine [89,129]. The solvent used for this purpose is mostly acetonitrile [89,120,129], although the application of internal standard previously prepared in acetonitrile is also described [104]. In this type of procedure agitation and centrifugation are almost always applied, in order to help the precipitation of proteins [89,104,120]. Associated with this technique is also described the application of simultaneously enzymatic hydrolysis [129]. Noble et al. [120], with 0.1 mL of sample were able to quantify 14 compounds, obtaining a recovery between 67 and 81, a detection limit between 0.0005 and 0.001 mg/kg and a quantification limit of 0.0005 mg/kg.

Another method used to prepare the sample is dilution. This procedure is mostly performed in urine [103,107,128,138,145], but is also described for serum [103,107]. The solutions used are acetate buffer [145] and water [128], agitation or centrifugation being also performed. It should be noted the work of Fleming et al. [128], with only 0.05 mL of urine, were able to quantify U-47700 with limits of detection and quantification of 1 ng/mL. Table 3 resumes the main analytical information about the published works that use protein precipitation and dilution as sample preparation techniques.

**Table 3.** Analytical information about protein precipitation and sample dilution as sample preparation techniques to determine NSO in biological specimens.

Analyte	Sample	Sample volume	Sample preparation information	Extraction conditions: solvent composition, temperature, agitation	Instrumental	LOD	LOQ	Recovery (%)	Reference
Acetylfentanyl, acetylnorfentayl, acrylfentanyl, butyrfentanyl, *p*-methoxybutyrfentanyl, *p*-chloroisobutyrfentanyl, despropionyl *p*-fluorofentanyl, furanyl fentanyl, norfentayl, ocfentanil, *o*-fluorofentanyl, valeryl fentanyl and *N*-methylcarfentanil	Whole blood	0.100 g	PP	Acetonitrile; 35ºC; agitation and centrifugation (10 min at 1 000 *g*)	UHPLC‐QTOF-MS	0.0005–0.001 mg/kg	0.0005 mg/kg	67–81	[120]
Acrylfentanyl	Blood	0.25 mL	PP	Acetonitrile; –; vortex (1 min) and centrifugation (3 min at 3 500 rpm)	GC-MS; UHPLC-MS/MS	0.05 ng/mL	0.10 ng/mL	–	[104]
Carfentanil, norcarfentanil	Blood (carfentanil, norcarfentanil) and urine (carfentanil)	0.25 mL	PP	Ice-cold acetonitrile (1 000 µL); 40ºC; shaking (5 min at 1 400 rpm) and centrifugation (20 min at 20 000 *g*)	LC-MS/MS	0.03 ng/mL	0.1 ng/mL	–	[89]
U-47700	Urine and blood	0.1 mL	PP and enzymatic hydrolysis (urine)	Acetonitrile (1:3); –; –	UHPLC-MS/MS	–	–	–	[129]
Acrylfentanyl, 4-chloroisobutyrfentanyl, 4-fluoroisobutyrfentanyl and tetrahydrofuranfentanyl	Urine and serum	–	Dilution	–	LC-MS/MS	<0.5 ng/mL	0.5 ng/mL	–	[103]
Butyrfentanyl, 4-fluorobutyrfentanyl	Serum and urine	–	Dilution	–	LC-MS/MS	–	0.5 ng/mL	–	[107]
Furanoylfentany	Urine	1 mL	Dilution	Acetate buffer (150 µL, pH = 9); –; centrifugation (5 min at 12 000 rpm)	LC-HRMS	–	–	–	[146]
U-47700	Urine	0.05mL	Dilution	Water (250 µL); room temperature; vortex	LC-QTOF	1 ng/mL	1 ng/mL	–	[154]
Acetylfentanyl	Urine	–	Dilution	n.s. (1:5); –; –	LC-MS/MS	≤ 0.1 ng/mL	–	–	[138]

NSO: novel synthetic opioids; LOD: limit of detection; LOQ: limit of quantitation; PP: protein precipitation; UHPL-QTOF-MS: ultra-high-performance liquid quadrupole time-of-flight mass spectrometry; –: undetected; GC-MS: gas chromatography mass spectrometry; UHPLC-MS/MS: ultra-high-performance liquid chromatography-tandem mass spectrometry; LC-MS/MS: liquid chromatography-tandem mass spectrometry; LC-HRMS: liquid chromatography-high-resolution mass spectrometry; LC-QTOF: liquid chromatography-quadrupole time-of-flight; n.s.: not specified.

Among the widely applied classic techniques used to determine NSO in biological specimens, liquid-liquid extraction continues to be the one of election in most toxicological analysis. Nevertheless, the large volumes of organic solvents that are required are considered, nowadays, a pitfall, hence some authors try to minimize these volumes, however assuring good extraction efficiencies. The minimum amount of solvent used for NSO determination is described by Jones et al. [129] who uses 0.5 mL of methanol to extract U-47700 from 100 µL of urine sample. However, methanol is not the most appropriate solvent for NSO extraction. Due to the alkaline nature of NSO, an alkaline extraction is the most reported for their quantification. This alkaline extraction most commonly employed uses 1-chlorobutane as extractant solvent for the specimen previously alkalinized with either ammonium hydroxide or buffers. The most described buffers are borate and phosphate buffers with pH ranging from 7.4 to 9. After this first extraction, most case reports describe a subsequent acid back-extraction for ultimate alkaline drug recovery. Hydrochloric acid (10 mmol/L to 3 mol/L), formic acid 0.1 mol/L and sulphuric acid 50 mmol/L are reported in this second extraction. The few extraction efficiencies reported in these cases are usually above 75% NSO dependent. Apart from this alkaline extraction followed by an acidic back extraction, other options were used with less time consume. Ethyl acetate and butyl acetate are also described in a considerable amount of case reports, with no extraction efficiencies shown by the authors. Also, diethyl ether was used to extract acetyl fentanyl from whole blood and urine specimens with a recovery ranging from 88% to 93%, and dichloromethane when applied to whole blood specimens gave an extraction efficiency of 76% for remifentanil. In both cases, the alkalinization of the sample was mandatory. A full review of the liquid-liquid extraction conditions reported for NSO is shown in Table 4.

**Table 4.** Liquid-Liquid extraction applied to biological specimens to determine NSO.

Analyte	Sample	Sample volume	LLE condtions						Instrumental	LOD	LOQ	Recovery (%)	Reference
			Pretreatment	Extraction solvent	Solvent volume	Extraction	Extraction time	Separation					
U-47700	Blood and urine	0.5 mL	1.5 mL distilled water and 250 μL concentrated ammonia	1-Chlorobutane	5 mL	Rotating extractor at 80 rpm	10 min	Centrifuged at 3 000 rpm for 15 min	LC-QTOF	0.8 ng/mL	n.s.	91	[131]
Acetyl fentanyl, β-hydroxythiofentanyl, butyryl fentanyl, carfentanil, furanyl fentanyl, MT-45 and U-47700	Whole blood	0.2 mL	400 μL pH 10 Tris-buffer	Methyl tert-butyl ether	1 mL	Pulse vortexing	∼30 s	Centrifuged for 5 min at ∼13 000 rpm	LC-MS/MS (n.s.)	n.s.	0.2 – 10 ng/mL	n.s.	[155]
Cyclopropylfentanyl and crotonylfentanyl	Blood	n.s.	Carbonate buffer (n.s.)	1-Chlorobutane	n.s.	n.s.	n.s.	n.s.	LC-MS	0.05 ng/mL	0.16 mg/mL	n.s.	[115]
Acetylfentanyl, carfentanil, valeryl fentanyl, acetylfentanyl-4-methylphenethyl, furanyl fentanyl, 3-methylfentanyl, butyryl fentanyl, fluorobutyryl fentanyl, acrylfentanyl, MT-45, W-19, AH-7921, U-47700, AH-8533, AH-8529, *para*-fluorofentanyl, ocfentanil, 4-methoxy butyryl fentanyl, β-hydroxy-thiofentanyl, IC-26	Urine	0.2 mL	Diluted with 0.8 mL 1.25 mol/L NaCl and 0.375% ammonium hydroxide in 50% isopropanol	90% MTBE/10% hexane	1 mL	Vortex mixing	n.s.	n.s.	LC-MS/MS (n.s.)	n.s.	0.1 – 0.4 ng/mL	82.5 – 95.5	[134]
Furanyl fentanyl and 4-ANPP	Blood and urine	1 mL (blood); 0.5 mL (urine)	(First extraction) 2 mL borate buffer (pH 9.0)	1-Chlorobutane	10 mL	Mixed by rotation	20 min	Centrifuged at 1 725 *g* for 10 min	GC-MS	n.s.	n.s.	n.s.	[87]
			(Second extraction) upper organic layer	3 mol/L HCl	0.4 mL	Mixed by rotation	15 min	Centrifuged at 1 725 *g* for 10 min					
			(Third extraction) aspirate and discard the upper layer, 100 µL saturated ammonium carbonate and 100 µL concentrated ammonium hydroxide	Chloroform	0.075 mL	Vortex mixing	n.s.	n.s.					
Acetyl fentanyl	Whole blood and urine	0.2 g	1 mL saturated NaCl, and 1 mL 1% Na_2_CO_3_	Diethyl ether	4 mL	Vortex mixing	2 min	Centrifugation at 4 000 rpm for 5 min	GC-MS/MS; LC-MS/MS-ESI+	n.s.	20 ng/g	88 – 93	[101]
Carfentanil	Whole blood	n.s.	Basic back extraction using sodium carbonate buffer	1-Chlorobutane	n.s.	n.s.	n.s.	n.s.	LC-MS/MS-ESI+	n.s.	0.05 ng/mL	n.s.	[113]
Acrylfentanyl	Whole blood	1 g	500 µL 1 mol/L Tris-buffer pH 11	Tert-butylmethylether	3 mL	Horizontal shaker	10 min	Centrifuged for 10 min at 4 000 *g*	LC-MS/MS-ESI+	n.s.	0.01 ng/g	84 – 94	[44]
Furanylfentanyl	Whole blood	2 g	501 µL 1 mol/L Tris-buffer pH 11	Tert-butylmethylether	3 mL	Horizontal shaker	10 min	Centrifuged for 10 min at 4 000 *g*	LC-MS/MS-ESI+	n.s.	0.1 ng/g	83 – 92	[31]
U-47700	Femoral blood, heart blood, urine, kidney, liver, lung and brain	n.s.	n.s.	1-Chlorobutane	n.s.	n.s.	n.s.	n.s.	LC-MS/MS (n.s.)	n.s.	n.s.	n.s.	[124]
U-47700	Whole blood and urine	1 mL	(First extraction) 0.1 mol/L sodium hydroxide (200 μL)	1-Chlorobutane	5 mL	Rotated	5 min	Centrifuged at 2 500 rpm for 2 min	LC-MS/MS-ESI+	n.s.	n.s.	n.s.	[127]
			(Second extraction) organic layer	1.0 mol/L formic acid	0.1 mL	Rotated	3 min	Centrifuged at 2 500 rpm for 2 min					
U-47700	Whole blood, liver, vitreous, urine and gastric	1 mL	(First extraction) 1mL deionized water; 1mL concentrated ammonium hydroxide	1-Chlorobutane	6 mL	mixed on a mechanical rocker	10 min	Centrifuged for 5 min at ∼2 400 *g*	GC-MS	∼5 ng/mL	20 ng/mL	n.s.	[125]
			(Second extraction) top organic layer	1.0 mol/L hydrochloric acid	2 mL	n.s.	10 min	Centrifuged for 5 min at ∼2 400 *g*					
			(Third extraction) remaining aqueous portion; 1 mL concentrated ammonium hydroxide	1-Chlorobutane	3 mL	n.s.	10 min	Centrifuged for 5 min at ∼2 400 *g*					
Carfentanil and furanyl fentanyl	Postmortem blood, vitreous and/or urine	2 mL	(First extraction) saturated borate buffer	THIA (78:20:2 mixture of toluene, hexane and isoamyl alcohol)	n.s.	n.s.	n.s.	n.s.	GC-MS	10 ng/mL	n.s.	n.s.	[108]
			(Second extraction) sulfuric acid and neutralization with NaHCO_3_/K_2_CO_3_	ethyl acetate	n.s.	n.s.	n.s.	n.s.					
Furanyl fentanyl and metabolites	Urine	0.1 mL	Incubated with glucuronidase from Red Abalone in acetate buffer, pH 5 at 37 °C for 1 h	90% acetonitrile/10% methanol	0.75 mL	n.s.	n.s.	n.s.	LC-(TOF)-MS and LC-MS/MS-ESI+	n.s.	≤0.5 ng/mL	n.s.	[28]
Ocfentanil	Whole blood, vitreous humor, mucous membrane of the nose, urine, stomach content, liver, kidney, brain tissue and bile	0.5 mL or 0.5 g	Kidney, liver, stomach content (semi-solid), bile and brain tissue were homogenized in water at a ratio (m/m) of 1:2 or 1:5 by means of Ultra Turrax; the swab of the mucous membrane of the nose was extracted in 0.5 mL ultrapure water; 1.0 mL potassium carbonate solution	*n*-Hexane: ethyl acetate (7:3, v/v)	5 mL	Vortex mixing	2 min	Centrifugation at 3 000 rpm for 5 min	LC-MS/MS-ESI+	2.1 ng/mL	2.1 ng/mL	87.6	[117]
Ocfentanil	Whole blood and urine	500 mg whole blood or 50 mg urine	0.9% sodium chloride solution in water was added resulting in 1 mL diluted sample	1-Chlorobutane	3 mL	Gently mixing the sample	n.s.	Centrifugation (10 min, 3 500 rpm)	LC-MS/MS-APCI	n.s.	2.5 ng/mL	n.s.	[36]
Acetyl fentanyl	Whole blood, liver, brain, vitreous humor, and urine	1.0 mL case blood or 1.0 g a 1:4 tissue homogenate	(First extraction) (n.s.) ammonium hydroxide	1-Chlorobutane	n.s.	n.s.	n.s.	n.s.	GC–MS	62.5 ng/mL	125 ng/mL	n.s.	[95]
			(Second extraction) (n.s.) acid back-extraction	n.s.	n.s.	n.s.	n.s.	n.s.					
U-47700	Blood and urine	n.s.	Back extraction using sodium carbonate buffer	1-Chlorobutane	n.s.	n.s.	n.s.	n.s.	LC-QTOF-MS/MS	≤78 ng/mL	≤312.5 ng/mL	n.s.	[64]
U-47700	Urine	0.1 mL	Hydrolisis	Methanol	0.5 mL	Vortex mixed	n.s.	n.s.	LC-MS/MS-ESI+	n.s.	n.s.	n.s.	[129]
U-47700	Urine	1 mL	Alkaline conditions (n.s.)	Methylene chloride:cyclohexane:isopropanol (4.5:4.5:1.0)	n.s.	n.s.	n.s.	n.s.	GC/MS/MS and LC-(TOF)-MS	n.s.	n.s.	n.s.	[130]
U-47700	Whole blood and urine	0.5 mL	β-Glucuronidase hydrolyzed urine; 1.0 mL 1 mol/L potassium carbonate	*n*-Hexane:ethyl acetate (7:3, v/v)	5 mL	Vortex mixing	2 min	Centrifugation at 3 000 rpm for 5 min	LC-MS/MS-ESI+	1.6 ng/mL	1.6 ng/mL	n.s.	[123]
U-47700	Blood and urine	0.5 mL	(First extraction) 0.2 mol/L Na_2_CO_3_ solution (0.5 mL, pH 10)	1-Chlorobutane	5 mL	Shaking	3 min	Centrifuging at 3 300 rpm for 3 min	HPLC-DAD	50 ng/mL	312.5 ng/mL	n.s.	[64]
			(Second extraction) supernatant	0.05 mol/L H_2_SO_4_	0.1 mL	Shaking	3 min	Centrifuging at 3 300 rpm for 3 min					
4-Fluorobutyrfentanyl	Blood and urine	2 mL	4 mL Tris-buffer (pH= 9), 4 mL acetonitrile, and ultrasonicated for 60 min	Ethyl acetate	10 mL	Shaking	120 min	Centrifuged (n.s.)	LC-MS and GC-MS	7 ng/mL	12 ng/mL	n.s.	[119]
4-Fluorobutyrfentanyl	Liver, kidney, brain, and stomach contents	5 g	10 mL aliquots of Tris-buffer (pH = 9) and 10 mL acetonitrile, and ultrasonicated for 60 min	Ethyl acetate	20 mL	Shaking	120 min	Centrifuged (n.s.)	LC-MS and GC-MS	7 ng/mL	12 ng/mL	n.s.	[119]
Acetyl fentanyl and butyryl fentanyl	Peripheral blood, heart blood, vitreous humor and urine	2 mL	(First extraction) (n.s.) saturated borate buffer	78:20:2 mixture of toluene, hexane and isoamyl alcohol	n.s.	n.s.	n.s.	n.s.	GC–MS	n.s.	n.s.	n.s.	[94]
			(Second extraction) top organic layer	sulfuric acid	n.s.	n.s.	n.s.	n.s.					
MT-45	Whole blood	0.5 mL	50 μL ammonium hydroxide	1-Chlorobutane/ acetonitrile (4:1, v/v)	4 mL	Rotated	n.s.	Centrifuged (n.s.) for 10 min	GC–MS	1 ng/mL	1 ng/mL	n.s.	[6]
Butyrfentanyl, carboxybutyrfentanyl, hydroxybutyrfentanyl, desbutyrfentanyl, norbutyrfentanyl	Heart blood, urine, muscle, liver, kidney, lung, spleen, adipose tissue, gastric content, frontal lobe, thalamus, cerebellum, centrum semiovale, praecuneus and hair	1 mL (blood), n.s (remaining specimens)	First extraction (pH 7.4)	Butyl acetate/ethyl acetate (1:1, v/v)	n.s.	n.s.	n.s.	n.s.	LC-MS/MS (n.s.)	n.s.	1 ng/mL	n.s.	[40]
			Second extraction (pH 13.5)	Butyl acetate/ethyl acetate (1:1, v/v)	n.s.	n.s.	n.s.	n.s.					
Acetyl fentanyl	Whole blood, liver, vitreous and urine	1 mL	1 mL deionized water; 1 mL concentrated ammonium hydroxide	1-Chlorobutane	6 mL	Mixed on a mechanical rocker	10 min	Centrifuged for 5 min at 2 400 *g*	GC–MS	50 ng/mL	100 ng/mL	n.s.	[34]
Butyr-fentanyl	Whole blood, liver, vitreous, urine and gastric	1 mL	(First extraction) 2 mL deionized water; 1 mL concentrated ammonium hydroxide	1-Chlorobutane	6 mL	Mixed on a mechanical rocker	10 min	Centrifuged for 5 min at 2 400 *g*	GC–MS	2 ng/mL	10 ng/mL	n.s.	[34]
			(Second extraction) top organic layer	1.0 mol/L hydrochloric acid	2 mL	Mixed on a mechanical rocker	10 min	Centrifuged for 5 min at 2 400 *g*					
			(Third extraction) remaining aqueous portion made alkaline with 1 mL concentrated ammonium hydroxide	1-Chlorobutane	3 mL	Mixed on a mechanical rocker	10 min	centrifuged for 5 min at 2 400 *g*					
AH-7921	Blood	0.5 mL	Borate buffer pH 11, 0.25 mL	Ethylacetate/heptane	1.2 mL	n.s.	n.s.	n.s.	n.s.	n.s.	n.s.	n.s.	[106]
AH-7921	Whole blood, urine, liver, kidney, spleen, heart, lung, brain, bile, gastric content	2 mL or 1 g	(First extraction) 2 mL deionized water; four drops concentrated potassium hydroxide (45%, w/v)	1-Chlorobutane	5 mL	Rotomixer	20 min	Centrifuged for 10 min at 3 000 rpm	GC–MS	n.s.	n.s.	n.s.	[53]
AH-7921	Blood and plasma	0.5 mL	(First extraction) 0.2 mol/L Na_2_CO_3_ solution (0.5 mL, pH 10)	1-Chlorobutane	5 mL	Shaking	3 min	Centrifuging at 3 300 rpm for 3 min	HPLC-DAD; LC-MS/MS-ESI+; LC-(TOF)-MS	n.s.	n.s.	n.s.	[156]
			(Second extraction) supernatant	0.05 mol/L H_2_SO_4_	0.1 mL	Shaking	4 min	Centrifuging at 3 300 rpm for 3 min					
3-Methylfentanyl	Blood (1); urine (2)	1 mL	(1) 400 μL Na_2_HPO_4_ buffer (pH 9); (2) hydrolyzed with 20 μL β-glucuronidase for 16 h 4 ºC , and adjusted to pH 7 with a few drops 1 mol/L HCl	Butyl acetate	0.6 mL	Vortex mixer	2 min	n.s.	LC-MS/MS-ESI+	n.s.	n.s.	n.s.	[49]
3-Methylfentanyl	Blood	1 mL	0.3 mL Tris-buffer (pH 11)	Butyl acetate	0.5 mL	Vortex mixer	2 min	n.s.	LC-MS/MS (n.s.)	0.1 ng/mL	n.s.	n.s.	[84]
Alfentanil, fentanyl, *p*-fluorofentanyl, *cis*-3-methylfentanyl, *trans*-3-methylfentanyl, α-methylfentanyl, norfentanyl, remifentanil and sufentanil	Blood (1); urine (2)	1 mL	(1) 400 μL Na_2_HPO_4_ buffer (pH 9); (2) hydrolyzed with 20 μL β-glucuronidase for 16 h 46ºC, and adjusted to pH 7 with a few drops 1 mol/L HCl	Butyl acetate	0.6 mL	Vortex mixer	2 min	Centrifuged (n.s.)	LC-MS/MS-ESI+	(1) ≤1 ng/mL; (2) ≤2 ng/mL	(1) ≤7 ng/mL; (2) ≤7 ng/mL	n.s.	[140]
Sufentanil	Serum	1 mL	(First extraction) 100 µL 1 mol/L NaOH	Toluene-2-propanol (10:1, v/v)	2 mL (0.2 mL)	Roller-shaker	20 min	Centrifugation at 4 000 *g* for 5 min	LC-MS/MS-ESI+	3 pg/mL	10 pg/mL	75	[157]
			(Second extraction) evaporated under vacuum and residue dissolved in 10 µL cyclo-Hexane	1 mol/L HCL	0.2 mL	Intense shaking	10 s	Centrifugation for 2 min at 4 000 *g*					
Remifentanil	Whole blood	1 mL	(First extraction) (n.s.)	Methanol	1 mL	Vortex mixer	5 s	Centrifuged for 15 min at 1 000 *g*	Capillary GC with nitrogen-specific detection	n.s.	0.2 ng/mL	79 – 85	[158]
			(Second extraction) (supernatant)	1-Chlorobutane	6 mL	Vortex mixer	5 s	Centrifuged for 10 min at 1 000 *g*					
			(Third extraction) (organic phase)	HCl (10 mmol/L)	0.2 mL	Vortex mixer	5 s	Centrifuged for 10 min at 1 000 *g*					
Remifentanil	Whole blood	0.5 mL	0.5 mL 0.1 mol/L phosphate buffer pH 7.4, the sample pH was readjusted back to pH 7	Dichloromethane	2 mL	Shaking mechanically	10 min	Centrifuged at 13 000 rpm for 10 min	LC-MS/MS-ESI+	n.s.	0.1 ng/mL	76	[159]
Alfentanil	Plasma	1 mL	(First extraction) 200 µL potassium hydroxide (0.5 mol/L)	Heptane:isoamyl alcohol, 98:2 v/v)	4 mL	Horizontally shaken mechanically at 2 Hz	10 min	Centrifuged at 420 *g* for 15 min	LC-UV/Vis	0.25 ng/mL	2 ng/mL	86.3	[160]
			(Second extraction) 3.8 mL the upper organic	Back extractant (0.5 mol/L KH_2_PO_4_, pH 2.8, adjusted with 85% H_3_PO_4_)	0.2 mL	Horizontally shaken mechanically at 2 Hz	10 min	Centrifuged at 420 *g* for 15 min					
Sufentanil	Blood, urine, vitreous humour, liver and kidney	2 mL	(First extraction) 1 mL 1.5 mol/L NaOH	hexane:ethanol (19:1 v:v)	5 mL	n.s.	n.s.	n.s.	GC-MS	n.s.	n.s.	n.s.	[161]
			(Second extraction) organic layer	O. 1 mol/L HCI	5 mL	n.s.	n.s.	n.s.					
			(Third extraction) aqueous layer and 1 mL 1.5 mol/L NaOH	hexane:ethanol	5 mL	n.s.	n.s.	n.s.					
α-Methylfentanyl	Liver	10 g	(First extraction) 40 mL water using a mechanical homogenizer; 3 mL 2 mol/L NaOH	hexane:ethanol (19:1)	60 mL	Mechanical shaker	10 min	n.s.	GC-MS	n.s.	n.s.	n.s.	[18]
			(Second extraction) organic layer	0.1 mol/L HCI	4 mL	Mechanical shaker	10 min	n.s.					
			(Third extraction) aqueous layer; 1 mL 2 mol/L NaOH	hexane:ethanol (19:1)	6 mL	Mechanical shaker	11 min	n.s.					

NSO: novel synthetic opioids; LLE: liquid-liquid extraction; LOD: limit of detection; LOQ: limit of quantitation; n.s.: not specified; LC-MS/MS-ESI+: liquid chromatography-tandem mass spectrometry with positive electrospray ionization; GC-MS: gas chromatography mass spectrometry; UHPL-QTOF-MS: ultra-high-performance liquid quadrupole time-of-flight mass spectrometry; GC-MS: gas chromatography mass spectrometry; LC-(TOF)-MS: liquid chromatography time-of-flight mass spectrometry; LC-MS/MS-APCI: liquid chromatography-tandem mass spectrometry with atmospheric pressure chemical ionization; LC-QTOF-MS/MS: liquid chromatography-quadrupole time-of-flight-tandem mass spectrometry; HPLC-DAD: high performance liquid chromatography-diode array detection; GC: gas chromatography; LC-UV/Vis: liquid chromatography with ultra-violet/visible.

The second most described pre-treatment technique is the solid phase extraction (SPE). SPE is known for presenting good compatibility with high throughput multiresidue analytical procedures and great extraction efficiencies associated. With this technique, many different cartridges with different sorbents can be used. Regarding NSO, if only one target analyte is meant to be determined, a reverse phase C_18_ sorbent is commonly applied. Examples of that is the use of this cartridges to determine U-47700 in serum and urine [132], and sufentanil in hair specimens [147] or sufentanil in plasma samples [148]. Also, hydrophilic and lipophilic cartridges have been applied to determine ramifentanil in plasma specimens showing extraction efficiencies above 90% [149].

Nevertheless, the most frequent analytical procedure involves a multitarget analyte extraction and determination [30,85,86,88,150]. A larger number of case reports about NSO apply mixed mode sorbents, such as PSCX or CleanScreen^®^ ZCDAU020 [88,150]. A wide range of recoveries are described for these sorbents, usually NSO and solvents applied dependent. However, they seem to be the chosen ones when it comes to a multi NSO determination. Also, a polymeric strong-cationic exchange cartridge, Strata X^®^, is described for acetylfentanyl and acetylnorfentanyl determination in urine samples [151]. The authors do not report any extraction recoveries with this sorbent. A full review of the solid phase extraction conditions reported for NSO is shown on Table 5.

**Table 5.** SPE applied to biological specimens to determine NSO.

Analyte	Sample	Sample volume/weight	SPE condtions					Instrumental	LOD	LOQ	Recovery (%)	Reference
			Sorbent	Pretreatment	Conditioning	Wash	Elution					
U-47700	Serum and urine	0.1 mL	Chromabond^®^	Urine samples were treated with β-glucuronidase/arylsulfatase for conjugate cleavage	n.s.	n.s.	n.s.	n.s.	n.s.	n.s.	n.s.	[132]
β-Hydroxythiofentanyl, butyryl/isobutyrylfentanyl, AH-7921, 2-furanylfentanyl, 4-ANPP, U-47700, MT-45, *para*-methoxybutyrylfentanyl, 4-methylphenethyl acetyl fentanyl, U-50488, acrylfentanyl, valerylfentanyl, carfentanil, *para*-fluorofentanyl, *ortho*-fluorofentanyl, *para*-fluorobutyrylfentanyl/FIBF and α-methylfentanyl	Blood, serum/plasma and urine	0.5 mL	Plexa PCX 3 mL/60 mg	Acetonitrile (1 mL) added to each sample for a protein precipitation step; 1 mL 0.1 mol/L phosphate buffer	2.0 mL acetonitrile; 2.0 mL deionized water	2.0 mL 0.1 mol/L hydrochloric acid and 2.0 mL acetonitrile	2.0 mL 5% ammonium hydroxide in acetonitrile	LC-MS/MS-ESI+	≤0.25 ng/mL	≤0.5 ng/mL	≥56	[88]
Furanylfentanyl, butyrylfentanyl, 4-ANPP, methoxyacetylfentanyl, THFF, FIBF, acrylfentanyl, *para*-fluorofentanyl, *ortho*-fluorofentanyl, carfentanil, β-methylfentanyl, isobutyrylfentanyl, *para*-methylfentanyl, cyclopentylfentanyl, cyclopropylfentanyl, β-hydroxyfentanyl and αmethylfentanyl	Blood	0.5 mL	130 mg CleanScreen^®^ DAU	2 mL phosphate buffer (pH 6)	3 mL methanol, 3 mL deionized water and 1 mL phosphate buffer	Deionized water (1.5 mL), 0.1 mol/L acetic acid (0.5 mL) and methanol	2 mL ethyl acetate/acetonitrile/ammonium hydroxide (78:20:2)	LC-MS/MS-ESI+	n.s.	n.s.	n.s.	[86]
U-47700 and furanyl fentanyl	Blood	0.5 mL	130 mg CleanScreen^®^ DAU	2 mL phosphate buffer (pH 6)	3 mL methanol, 3 mL deionized water and 1 mL phosphate buffer	(1.5 mL)	2 mL ethyl acetate/acetonitrile/ammonium hydroxide (78:20:2)	LC-MS/MS (n.s.)	n.s.	n.s.	n.s.	[162]
Acetyl fentanyl and acetyl norfentanyl	Urine	0.08 mL	Strata X-B 33 μ, 30 mg/3 mL	310 μL 0.1 mol/L sodium acetate buffer (pH 5.0) containing β-glucuronidase and incubating for 60 min at 37 °C	n.s.	1 mL a 0.1% formic acid aqueous solution and 1 mL a 70:30 water/methanol solution	Twice with 0.5 mL a basic 50:50 methanol/acetonitrile solution (2% ammonium hydroxide)	LC-MS/MS-ESI+	0.5 – 1 ng/mL	1.06 – 1.62 ng/mL	n.s.	[151]
*Cis-* and *trans*-3-methylfentanyl	Blood	0.5 mL	130 mg CleanScreen^®^ DAU	2 mL phosphate buffer (pH 6)	3 mL methanol, 3 mL deionized water and 1 mL phosphate buffer	Deionized water (1.5 mL), 0.1 mol/L acetic acid (0.5 mL) and methanol	n.s.	LC-QTOF-MS	n.s.	0.1 ng/mL	n.s.	[163]
Acrylfentanyl	Whole blood	0.25 mL	UCT CleanScreen^®^ CSDAU020	3 mL 0.1 mol/L phosphate buffer (n.s)	5 mL hexane, 3 mL methanol, 3 mL deionized water and 1mL 1.0 mol/L acetic acid	0.1 mol/L phosphate buffer and 1.0 mol/L acetic acid	(n.s.) 50:50 ethyl acetate:hexane and 78:20:2 methylene chloride:isopropanol:ammonium hydroxide	LC-MS/MS-ESI+	0.05 ng/mL	0.10 ng/mL	n.s.	[104]
Carfentanil, 3-methylfentanyl, 2-furanyl fentanyl and acetyl fentanyl	Whole blood	2 mL	200 mg CleanScreen^®^ ZSDAU020	0.25 mL acetonitrile were vortexed for 7 s, then 2 mL water and vortexed again for 7 s; 3 mL 0.1 mol/L phosphate buffer (pH 6.0)	3 mL methanol, followed by 2 mL deionized water, and 1.5 mL phosphate buffer (pH 6.0)	2 mL deionized water, 1.5 mL 1 mol/L acetic acid, and 2 mL methanol	3 mL dichloromethane/2-propanol/ammonium hydroxide (78:20:2)	LC-MS/MS(n.s.)	n.s.	0.10 ng/mL	n.s.	[85]
*Ortho*-fluorofentanyl	Serum, blood and urine	0.2 mL	HybridSPE^®^-PLus 96-well plates	Precipitated with the addition of acetonitrile	n.s.	n.s.	n.s.	LC-QTOF-MS	≤0.5 ng/mL	≤1 ng/mL	n.s.	[118]
*para*-fluoroisobutyryl fentanyl, butyryl fentanyl, furanyl fentanyl and carfentanil and U-47700	Whole blood, liver, brain and urine	1 mL or 1 g	CleanScreen^®^ ZSDAU020	Buffered with 0.1 mol/L, pH 6 sodium phosphate (4 mL)	Methanol (3 mL), deionized water (3mL), and 0.1 mol/L, pH 6 sodium phosphate buffer (1mL)	Deionized water (3 mL) and 1 mol/L acetic acid (1 mL); hexane (2 mL), hexane:ethyl acetate (50:50, 3 mL), and methanol (3 mL)	Dichloromethane:isopropanol:ammonium hydroxide (78:20:2, 3 mL).	LC-Ion Trap-MSn	0.2 – 0.5 ng/mL	n.s.	n.s.	[150]
U-47700, U-50488, furanyl fentanyl	Whole blood	0.5 mL	130 mg CleanScreen^®^ DAU	2 mL phosphate buffer (pH 6.0)	3 mL methanol, 3 mL deionized water and 1 mL phosphate buffer	1.5 mL deionized water, 0.5 mL 0.1 mol/L acetic acid and 1.5 mL methanol	2 mL ethyl acetate/acetonitrile/ ammonium hydroxide (78:20:2)	LC-MS/MS-ESI+	0.5 ng/mL	1 ng/mL	n.s.	[30]
Acetyl fentanyl	Cardiac blood from the right ventricle, gastric contents or urine	1.0 mL	Waters OASIS HLB C18	n.s.	1 mL methanol; 1 mL distilled water	(n.s.) Methanol (5%) in water	Methanol	GC-MS and LC-MS/MS-ESI+	n.s.	n.s.	n.s.	[93]
Acetyl fentanyl, acetyl norfentanyl and butyryl fentanyl	Peripheral blood, heart blood, vitreous humor, gastric contents, brain, liver, bile and urine	1.0 mL or 1.0 g	SPEC MP3 SPE columns	Bile, gastric contents and urine were diluted 1:10 with deionized water; brain and liver tissue were diluted with 6.0 g deionized water; 1 mL pH 6.0 phosphate buffer	0.4 mL methanol; 0.4 mL 100 mmol/L phosphate buffer (pH 6)	0.4 mL deionized water; 0.4 mL 100 mmol/L acetic acid	1 mL 78:20:2 dichloromethane/isopropanol/ammonia (v:v:v)	LC-MS/MS-(n.s.)	n.s.	1 ng/mL	n.s.	[94]
U-47700	Urine	0.25 mL	PSCX cartridges	25 μL β-glucuronidase and 425 μL 0.1 mol/L sodium acetate buffer; incubated at 60 °C for 45 min	n.s.	0.5 mL water, 0.2 mL 100 mmol/L HCl, 0.1 mL water:methanol (75:25 v:v)	250 μL ethyl acetate:methanol:ammonium hydroxide (78:20:2 v:v:v)	LC-MS-ESI+	1 ng/mL	1 ng/mL	n.s.	[154]
Alfentanil	Serum and blood	0.2 mL	Bakerbond SPE C18 (3 mL, 500 mg)	6 mL phosphate buffer (0.1 mol/L, pH 6)	2 × 3 mL methanol and 2 mL water	1 mL 0.1 mol/L acetic acid	3 × 1 mL dichloromethane/acetone (50:50 v/v); 3 × 1 mL dichloromethane/2-propanol/ammonium hydroxide (40:10:2 v/v/v)	LC-MS-ESI+	0.3 ng/mL	1 ng/mL	49 – 60	[122]
Norfentanyl, acetyl fentanyl and acetyl norfentanyl	Postmortem heart blood, peripheral blood, bile, brain, liver, urine and vitreous humor	1.0 mL or 1.0 g	SPEC MP3 SPE columns	1 mL pH 6.0 phosphate buffer	0.4 mL methanol; 0.4 mL 100 mmol/L phosphate buffer (pH 6)	0.4 mL deionized water; 0.4 mL 100 mmol/L acetic acid	1 mL 78:20:2 dichloromethane/isopropanol/ammonia (v:v:v)	LC-MS/MS-(n.s.)	n.s.	≤1 ng/mL	94 – 118	[92]
AH-7921	Urine	2 mL	CleanScreen^®^ ZCDAU020	3 mL 0.1 mol/L phosphate buffer (pH 6.0)	3 mL methanol, 3 mL deionized water and 2 mL 0.1 mol/L phosphate buffer (pH 6.0)	(First wash) 2 mL deionized water, 2 mL 20% acetonitrile in deionized water and 2 mL 0.1 mol/L acetic acid	3 mL dichloromethane/isopropanol/ammonium hydroxide (78:20:2)	GC-MS	n.s.	n.s.	n.s.	[53]
						(Second wash) 2 mL hexane followed by 3 mL methanol						
Remifentanil	Plasma	0.25 mL	Waters OASIS HLB C18	Acidified with 5 µL phosphoric acid (85%) and diluted to 500 µL with water	1 mL methanol, 2 mL water	(n.s.) water containing 2% acetic acid; 1 mL water containing 5% methanol and 2% acetic acid	1 mL methanol 60% containing 2% acetic acid	LC-MS-ESI+	0.18 ng/mL	0.5 ng/mL	98.4 – 112.9	[149]
Norfentanyl, carfentanil, sufentanil, norsufentanil, norcarfentanil, lofentanil, 3-methylfentanyl, α-methylfentanyl, ohmefentanyl, remifentanil and remifentaniI-COOH	Urine	0.5 mL	1 mL Waters OASIS HLB C18	0.5 mL 0.2 mol/L acetate buffer (pH 4.0) equilibrated for 30 min	1 mL methanol, 1 mL water	(n.s.) 20% methanol in water	1 mL methanol	LC-MS/MS-ESI+	3 – 27 pg/mL	n.s.	10 – 87	[164]
Sufentanil	Plasma	1 mL	Baker, C18, 200 mg	n.s.	3.0 mL methanol; 1.0 mL KOH (1 mol/L); 1.0 mL water and 0.2 mL ethanol /water (5:95, v/v)	0.7 mL K_2_HPO_4_ (10 mmol/L) and 1.4 mL water	1.4 mL methanol	LC-MS/MS-ESI+	n.s.	0.3 ng/mL	89.9	[148]
Sufentanil	Hair	50 mg	Chromabond 18 ec	Incubated in 2 mL Soerensen buffer of pH 7.6 for 2 h at 40 °C	n.s.	n.s.	3 times with acetone/dichlormethane (3:1)	GC-MS/MS	n.s.	n.s.	n.s.	[165]

NSO: novel synthetic opioids; SPE: solid phase extraction; LOD: limit of detection; LOQ: limit of quantitation; LC-MS/MS-ESI+: liquid chromatography-tandem mass spectrometry with positive electrospray ionization; LC-QTOF-MS: liquid chromatography-quadrupole time-of-flight mass spectrometry; GC-MS: gas chromatography mass spectrometry; LC-MS-ESI+: liquid chromatography-mass spectrometry with positive electrospray ionization.

### New approaches of sample preparation to determine NSO in biological specimens

Nowadays there are new trends relative to sample preparation techniques. The concern for the use of “greener” extraction techniques, particularly those that use low amounts of organic solvents and sample, minimizing the solvent residues (with obvious environmental advantages) and finally the possibility of reusing the extraction device (in some of the techniques) as well as the possibility of its automation has led to an effort of researchers to design and develop new operational paradigms. At present, researchers are following a new trend in the use of miniaturized or microtechnical, since the more traditional approaches of LLE and SPE use considerable amounts of organic solvents. Examples of these new paradigms are the use of dried blood spots (or dried matrix spots), molecular printing polymers, solid phase microextraction, liquid-liquid microextraction or QuEChERS [152]. About this type of techniques and their application in the determination of NSO, there are few reports in the literature, with only two publications that use QuEChERS and no other type of miniaturized systems. This circumstance is probably due to the fact that the boom of intoxication cases with this type of compounds is relatively recent and the researchers did not have enough time to delineate and design new methodologies. Concerning the use of QuEChERS, Yonemitsu et al. [97] quantified acetylfentanyl and 4-methoxy PV8 using ultra-high-performance liquid chromatography tandem mass spectrometry (UHPLC-MS/MS) equipment. In this work, 0.5 mL of blood, urine and gastric contents were used, which were treated with anhydrous magnesium sulphate (6 g) and anhydrous sodium acetate (1.5 g), followed by vigorous shaking (30 s) and centrifugation (10 min at 1 500 *g*). Recoveries between 55% and 80% were obtained. In another study performed by Usui et al. [116], MT-45 was quantified using PESI-MS/MS equipment. For this, 1.5 mL of deionized water, 1 mL of acetonitrile, 5 stainless beads and 0.5 g of pre-packed extraction packet (6 g of magnesium sulphate and 1.5 g of sodium acetate) were added to 0.5 g of liver, brain, heart, lung and kidney samples. Then, this mixture was vigorously shaken by a bead-type homogenize at 2 500 rpm/min for 30 s and centrifuged at 3 000 *g* for 1 min. These authors did not present the limits of detection, limits of quantification nor percentages of recovery.

## Conclusion

Among the variety of articles reviewed, including case reports, it is notable that lethal doses for NPS are often variable and deaths associated with these compounds seem to occur at both low and high concentrations, probably due to different degrees of tolerance for different individuals. In most cases, NSO are found to be combined with other psychoactive substances, such as synthetic cathinones, synthetic cannabinoids, antidepressants, antipsychotics, as well as caffeine and acetaminophen. Common autopsy findings are pulmonary congestion and cerebral oedema.

When a substance is scheduled by DEA or EMCDDA, it does not seem to decrease the illicit traffic of NSO. It seems that as soon as one substance is schedule, a new analogue occurs on the market, as a respond to users, who keep searching for alternatives. This is the case of U-47700 which is an analogue of AH-7921 and was immediately replaced with U-49900, after being scheduled.

Individuals who suffer NSO intoxications are often unaware of the real constitution of the drug they bought and consumed. Users might think they were consuming heroin, fentanyl or oxycodone, when, in fact, they were consuming one NSO, which might have a higher potency, increasing the liability of overdose. Clinicians dealing with fentanyl intoxication cases should consider that it could, in fact, be a fentanyl analogue.

Given the growing awareness about NSO and the wide number of fatalities reported within the last few years, it is an important task to accurately identify these compounds in biologic matrices. This accurate identification may allow an effective treatment and reverse the respiratory depression. In addition, it will facilitate the gathering of epidemiologic data to timely inform public health and law enforcement authorities. A variety of analytical methods and techniques have been used to determine these compounds; most of which are based on LC/MS/MS allowing a maximum sensitivity and the possibility of new metabolites identification. In fact, because the toxicokinetic of these new compounds is mostly unknown, the discovery of their metabolites, as well as the creation of own libraries is very important. Nevertheless, in many cases there are no reference standards available.

Therefore, procedures involving for instance HRMS, TOF or Orbitrap present several advantages concerning this issue. Furthermore, developing new immunoassay techniques allowing adequate screening of these compounds is undoubtedly relevant, as many laboratories cannot afford expensive chromatographic systems (for example, at hospital emergency services). In addition, special attention should be given to efficient sample preparation procedures, mainly new miniaturized techniques. For this reason, it would be very helpful to include synthetic opioids in the routine toxicological screening procedures, including analysis in alternative specimens (such as hair analysis), if available, to investigate poly-drug use and possible tolerance to opioids.

Finally, the new trend is represented by fast, sensitive and specific routes, which are also miniaturized and prone to automation. Analytical methodologies should be developed to identify these compounds in cases of intoxication before and after death, validated before routine application, and analytical data should be shared between different communication platforms. To address this public health problem, better international collaboration, effective legislation, effective investigation and control of suspicious “research chemicals” online forums and continuous community alertness are required.

## Supplementary Material

Supplemental Material
