# Supplementary material for: Novel synthetic opioids – toxicological aspects and analysis
Source: Forensic Sci Res. 2019 Jul 3;4(2):111–40. doi: 10.1080/20961790.2019.1588933 (PMC6609355; doi:10.1080/20961790.2019.1588933)
Supplement: Supplemental Material [file TFSR_A_1588933_SM6538.docx]

**Table 1 – Supplementary material** - Information about the concentrations found of NSO in cases of intoxication.

|  | **Concentrations** | | | | | | | | | | | | | | | | | | **References** |
| --- | --- | --- | --- | --- | --- | --- | --- | --- | --- | --- | --- | --- | --- | --- | --- | --- | --- | --- | --- |
| **Compounds** | **BL** | **PF** | **CF** | **S** | **U** | **VH** | **H** | **B I** | **H** | **BR** | **LI** | **K** | **LU** | **M** | **Ad T** | **G** | **SP** | **NS** |  |
| **2,2'-Difluorofentanyl** | - | - | - | - | - | - | - | - | - | - | - | - | - | - | - | - | - | - | - |
| **3-methylfentanyl or cis-3-methylfentanyl or trans-3-methylfentanyl** | 0.3-0.9 (L) (*post-mortem*) | - | - | - | - | - | - | - | - | - | - | - | - | - | - | - | - | - | [84] |
|  | 0.06  -3.24 (L) (*post-mortem*) | - | - | - | 0.02-27.50 (L) | - | - | - | - | - | - | - | - | - | - | - | - | - | [49] |
|  | pBL: 1.7 (L)  hBL: 2.6 (L) (*post-mortem*) | - | - | - | - | 0.65 (L) | - | - | - | - | - | - | - | - | - | - | - | - | [85] |
|  | - | - | - | - | 0.29-21.50 (Pm) | - | - | - | - | - | - | - | - | - | - | - | - | - | [134] |
|  | 0.11-3.43 (L) (*post-mortem*) | - | - | - | - | - | - | - | - | - | - | - | - | - | - | - | - | - | [86] |
| **3-methylthiofentanyl** | - | - | - | - | - | - | - | - | - | - | - | - | - | - | - | - | - | - | - |
| **4-ANPP or 4-anilino-N-phenethylpiperidine** | - | - | - | - | 0.35-372.72 (Pm) | - | - | - | - | - | - | - | - | - | - | - | - | - | [28] |
|  | pBL:4.3 (L)  hBL:5.8 (L)  (*post-mortem*) | - | - | - | Positive | <0.20^a^ | - | - | - | - | >40 (L) | - | - | - | - | Negative | - | - | [87] |
|  | 0.1-410 (L) (*post-mortem*) | - | - | - | - | - | - | - | - | - | - | - | - | - | - | - | - | - | [88] |
|  | 8.7 (L) (*post-mortem*) | - | - | - | - | - | - | - | - | - | - | - | - | - | - | - | - | - | [89] |
|  | 0.53 (L) (*post-mortem*) | - | - | - | - | - | - | - | - | - | - | - | - | - | - | - | - | - | [89] |
| **4-Methoxybutyrylfentanyl or 4-Methoxybutyrfentanyl** | - | - | - | 1.3-11 (T) | 15.8-1000 (T) | - | - | - | - | - | - | - | - | - | - | - | - | - | [90] |
|  | 79 (L) (*post-mortem*) | - | - | - | - | - | - | - | - | - | - | - | - | - | - | - | - | - | [88] |
| **Acetyl-alpha-methylfentanyl or alpha-acetylmethylfentanyl** | - | - | - | - | - | - | - | - | - | - | - | - | - | - | - | - | - | - | - |
| **Acetyldihydrocodeine** | - | - | - | - | - | - | - | - | - | - | - | - | - | - | - | - | - | - | - |
| **Acetyl fentanyl or Acetylfentanyl** | pBL: 2-600 (L) (*post-mortem*) | - | - | - | - | - | - | - | - | - | - | - | - | - | - | - | - | - | [91] |
|  | pBL:6-600 (L)  hBL:2-980 (L) (*post-mortem*) | - | - | - | 3-16000 (L) | 1-900 (L) | - | 22-4800 (L) | - | 21-1700 (L) | 29-1900 (L) | - | - | - | - | - | - | - | [92] |
|  | 0.65 (L) (*post-mortem*) | - | - | - | - | - | - | - | - | - | - | - | - | - | - | - | - | - | [30] |
|  | pBL:260 (L)  hBL: 250 (L) (*post-mortem*) | - | - | - | 2,600 (L) | 240 (L) | - | - | - | - | 1 (L) | - | - | - | - | - | - | - | [34] |
|  | - | - | - | 0.6-51.6 (T) | 2.4-3180 (T) | - | - | - | - | - | - | - | - | - | - | - | - | - | [90] |
|  | 270 (L) (*post-mortem*) | - | - | - | - | - | - | - | - | - | - | - | - | - | - | - | - | - | [93] |
|  | pBL: 38 (L)  hBL: 32 (L) (*post-mortem*) | - | - | - | 540 (L) | 38 (L) | - | - | - | - | 110 (L) | - | - | - | - | <70 mg (L) | - | - | [39] |
|  | pBL: 21 (L)  hBL: 95 (L) (*post-mortem*) | - | - | - | 8 (L) | 68 (L) | - | 330 (L) | - | 200 (L) | 160 (L) | - | - | - | - | 28,000 (L) | - | - | [94] |
|  | pBL: 192-255 (L)  hBL: 210-285 (L) (*post-mortem*) | - | - | - | 2,720-3,420 (L) | 140 (L) | - | - | - | 620 (L) | 1,100 (L) | - | - | - | - | - | - | - | [95] |
|  | iBL: 235 (L) (*post-mortem*) | - | - | - | 234 (L) | 131 (L) | - | - | - | - | 2400 (L) | - | - | - | - | - | - | - | [96] |
|  | pBL: 153 (L)  hBL:239 (L) (*post-mortem*) | - | - | - | 240 (L) | - | - | - | - | - | - | - | - | - | - | 880 (L) | - | - | [97] |
|  | 0.01-5.00 ng/g (L) (*post-mortem*) | - | - | - | - | - | - | - | - | - | - | - | - | - | - | - | - | - | [44] |
|  | pBL: 2.2 (L)  hBL: 7.2 (L) (*post-mortem*) | - | - | - | - | 1.3 (L) | - | - | - | - | - | - | - | - | - | - | - | - | [85] |
|  | fBL: 440 (L) (*post-mortem*) | - | - | - | - | - | - | - | - | - | - | - | - | - | - | - | - | - | [98] |
|  | 0.31 (L) (*post-mortem*) | - | - | - | - | - | - | - | - | - | - | - | - | - | - | - | - | - | [99] |
|  | - | - | - | - | 2.4-3178 (Ic) | - | - | - | - | - | - | - | - | - | - | - | - | - | [100] |
|  | - | - | - | - | 0.10-91.00 (Pm) | - | - | - | - | - | - | - | - | - | - | - | - | - | [134] |
|  | BL:520 (L)  pBL: 0.13-0.45 (L)  fBL: 1.9-240 (L)  hBL: 0.43-2100 (L)  sBL: 1.6-220 (L)  (*post-mortem*) | - | - | - | - | - | - | - | - | - | - | - | - | - | - | - | - | - | [101] |
|  | pBL: 125 ng/g (L)  hBL: 155 ng/g (L)  (*post-mortem*) | - | - | - | 570 ng/g (T) and 126 ng/g (L) | - | - | - | - | - | - | - | - | - | - | - | - | - | [102] |
|  | pBL: 0.1-37 (L) (*post-mortem*) | - | - | - | - | - | - | - | - | - | - | - | - | - | - | - | - | - | [103] |
| **Acetyl norfentanyl** | pBL: 1-86 (L) (*post-mortem*) | - | - | - | - | - | - | - | - | - | - | - | - | - | - | - | - | - | [91] |
|  | pBL: 1-63 (L)  hBL:1-86 (L) (*post-mortem*) | - | - | - | 17-18000 (L) | 1-25 (L) | - | 13-1400 (L) | - | 4-64 (L) | 4-190 (L) | - | - | - | - | - | - | - | [92] |
|  | pBL: <1 (L)  hBL: 1.2 (L) (*post-mortem*) | - | - | - | <1 (L) | <1 (L) | - | 4 (L) | - | <4 (L) | <4 (L) | - | - | - | - | 8.9 (L) | - | - | [91] |
| **Acrylfentanyl or N-phenyl-N-[1-(2-phenethyl)piperidin-4-yl]prop-2-enamide** | - | - | - | 0.5-2.1 (T) | 1.8-196 (T) | - | - | - | - | - | - | - | - | - | - | - | - | - | [104] |
|  | 0.64-2.1 (L) (*post-mortem*) | - | - | - | - | - | - | - | - | - | - | - | - | - | - | - | - | - | [99] |
|  | 0.1-29 (L) (*post-mortem*) | - | - | - | - | - | - | - | - | - | - | - | - | - | - | - | - | - | [88] |
|  | - | - | - | - | 2.0-196 (Ic) | - | - | - | - | - | - | - | - | - | - | - | - | - | [100] |
|  | - | - | - | - | 0.18-95.37 (Pm) | - | - | - | - | - | - | - | - | - | - | - | - | - | [134] |
|  | pBL: 0.3-0.95 (L) (*post-mortem*) | - | - | - | - | - | - | - | - | - | - | - | - | - | - | - | - | - | [105] |
| **AH-7921** | pBL: 0.03-0.99 mg/g (L) (*post-mortem*) | - | - | - | - | - | - | - | - | - | - | - | - | - | - | - | - | - | [106] |
|  | pBL: 330-430 (L) (*post-mortem*) | - | - | - | - | - | -- | - | - | - | - | - | - | - | - | - | - | - | [107] |
|  | pBL: 9100 (L)  hBL: 3900 (L) (*post-mortem*) | - | - | - | 6000 (L) | - | - | 17000 (L) | 5100 (L) | 7700 (L) | 26000 (L) | 7200 (L) | 21000 (L) | - | - | 120 mg/125 mL (L) | 8000 (L) | - | [53] |
|  | pBL: 50-4460 (L)  (*post-mortem*) | - | - | - | - | - | - | - | - | - | - | - | - | - | - | - | - | - | [108] |
|  | pBL: 450 (L)  hBL: 480 (L)  (*post-mortem*) | 480 (L) | - | - | 760 (L) | 190 (L) | - | - | - | - | 530 µg/L (L) | - | - | - | - | 40000 (L) | - | - | [52] |
| **Alfentanil** | <0.8 (L) (*post-mortem*) | - | - | - | - | - | - | - | - | - | - | - | - | - | - | - | - | - | [89] |
| **Alpha-methylfentanyl** | 3.1 (L) (*post-mortem*) | - | - | - | - | - | - | 6.4 (L) | - | - | 78 (L) | - | - | - | - | - | - | - | [18] |
| **Alpha-methylthiofentanyl** | - | - | - | - | - | - | - | - | - | - | - | - | - | - | - | - | - | - | - |
| **Anilino-N-phenethylpiperidine-SO_4_ or ANPP-SO_4_** | - | - | - | - | 0.3-600 (Pm) | - | - | - | - | - | - | - | - | - | - | - | - | - | [28] |
| **Beta-hydroxyfentanyl** | - | - | - | - | - | - | - | - | - | - | - | - | - | - | - | - | - | - | - |
| **Beta-hydroxy-3-methylfentanyl** | - | - | - | - | - | - | - | - | - | - | - | - | - | - | - | - | - | - | - |
| **Beta-hydroxythiofentanyl** | - | - | - | - | - | - | - | - | - | - | - | - | - | - | - | - | - | - | - |
| **Butyrfentanyl or Butyrylfentanyl** | - | - | - | 0.6-0.9 (T) | 2.0-65.6 (T) | - | - | - | - | - | - | - | - | - | - | - | - | - | [109] |
|  | 0.33-26 (L) (*post-mortem*) | - | - | - | - | - | - | - | - | - | - | - | - | - | - | - | - | - | [30] |
|  | pBL: 66 (L)  hBL: 39 (L)  (*post-mortem*) | - | - | - | 1100 (L) | - | 11,000 (L) | - | - | 200-340 (L) | 57 (L) | 160 (L) | 3100 (L) | 110 (L) | 550 (L) | 2000 (L) | 590 (L) | - | [40] |
|  | pBL: 58 (L)  hBL: 97 (L) (*post-mortem*) | - | - | - | 670 (L) | 40 (L) | - | - | - | - | 320 (L) | - | - | - | - | 170 mg (L) | - | - | [39] |
|  | pBL: 3.7-99 (L)  hBL: 9.2-220 (L)  (*post-mortem*) | - | - | - | 2-64 (L) | 9.8-32 (L) | - | 49-260 (L) | - | 63-93 (L) | 39-41 (L) | - | - | - | - | 4,000-590 (L) | - | - | [94] |
|  | 0.1-760 (L) (*post-mortem*) | - | - | - | - | - | - | - | - | - | - | - | - | - | - | - | - | - | [88] |
|  | - | - | - | - | 192 (Ic) | - | - | - | - | - | - | - | - | - | - | - | - | - | [100] |
|  | - | - | - | - | 0.29-110.41 (Pm) | - | - | - | - | - | - | - | - | - | - | - | - | - | [134] |
| **Carfentanil** | hBL: 0.12-1.3 (L)  (*post-mortem*) | - | - | - | - | - | - | - | - | - | - | - | - | - | - | - | - | - | [110] |
|  | 0.41-1.4 (T) (ante-mortem) | - | - | - | - | - | - | - | - | - | - | - | - | - | - | - | - | - | [111] |
|  | pBL: 0.0104-0.529 (L)  hBL: 0.241 (L)  iBL: 0.0233-0.234 (L)  sBL:0.0301-0.617 (L)  (*post-mortem*) | - | - | - | - | - | - | - | - | - | - | - | - | - | - | - | - | - | [112] |
|  | pBL: 0.33 (DUID)  fBL: 0.36 (L)  hBL: 1.9 (L) (*post-mortem*) | - | - | - | - | - | - | - | - | - | - | - | - | - | - | - | - | - | [84] |
|  | - | - | - | 0.6 (T) | 1.3 (T) | - | - | - | - | - | - | - | - | - | - | - | - | - | [113] |
|  | 0.1-120 (L) (*post-mortem*) | - | - | - | - | - | - | - | - | - | - | - | - | - | - | - | - | - | [88] |
|  | 0.52 (T) (ante-mortem) | - | - | - | - | - | - | - | - | - | - | - | - | - | - | - | - | - | [114] |
|  | 92 (L) (*post-mortem*) | - | - | - | 2.8 (L) | 23 (L) | - | - | - | - | - | - | - | - | - | - | - | - | [115] |
|  | pBL: 0.22-3.3 (L)  hBL: 0.57 (L) aBL: 1.05 (L)  (*post-mortem*) | - | - | - | - | - | - | - | - | - | - | - | - | - | - | - | - | - | [116] |
|  | 0.021–4.004 (L) (*post-mortem*) | - | - | - | 0.03–12.163 (L) | - | - | - | - | - | - | - | - | - | - | - | - | - | [117] |
| **Cyclopropylfentanyl** | pBL: 1.4-43.3 (L)  fBL: 3.3-23.2 (L)  hBL: 11.6 (L)  iBL: 6.8-42.8 (L)  ahBL: 8.5 (L) (*post-mortem*) | - | - | - | - | - | - | - | - | - | - | - | - | - | - | - | - | - | [99] |
|  | pBL: 16.6-28.9 (L)  (*post-mortem*) | - | - | - | - | - | - | - | - | - | - | - | - | - | - | - | - | - | [118] |
| **Despropionylmethylfentanyl** | 3.8 (L) (*post-mortem*) | - | - | - | - | - | - | 12.7 (L) | - | - | 5.7 (L) | - | - | - | - | - | - | - | [18] |
| **Furanylfentanyl or furanyl fentanyl or 2-furanylfentanyl** | pBL: 6.1-6.2 (L)  fBL: 12.9 (L)  aBL: 2.5-76 (L) (*post-mortem*) | - | - | - | - | - | - | - | - | - | - | - | - | - | - | - | - | - | [30] |
|  | - | - | - | 4.4-148 (T) | 179-1430 (T) | - | - | - | - | - | - | - | - | - | - | - | - | - | [90] |
|  | hBL: 0.34 (L) (*post-mortem*) | - | - | - | - | - | - | - | - | - | - | - | - | - | - | - | - | - | [110] |
|  | 0.17-1.1 (T) (ante-mortem) | - | - | - | - | - | - | - | - | - | - | - | - | - | - | - | - | - | [111] |
|  | pBL: 0.61 (L)  (*post-mortem*) | - | - | - | - | - | - | - | - | - | - | - | - | - | - | - | - | - | [112] |
|  | pBL: 5.5 (L)  hBL: 8.7 (L)  (*post-mortem*) | - | - | - | - | 30 (L) | - | - | - | - | - | - | - | - | - | - | - | - | [84] |
|  | - | - | - | - | 0.26-392.32 (Pm) | - | - | - | - | - | - | - | - | - | - | - | - | - | [28] |
|  | pBL: 1 (L)  (*post-mortem*) | - | - | - | - | - | - | - | - | - | - | - | - | - | - | - | - | - | [98] |
|  | pBL: 0.38-2.74 ng/g (L) (*post-mortem*) | - | - | - | - | - | - | - | - | - | - | - | - | - | - | - | - | - | [31] |
|  | 0.52 (L) (*post-mortem*) | - | - | - | - | - | - | - | - | - | - | - | - | - | - | - | - | - | [99] |
|  | pBL: 1.9 (L)  hBL: 2.8 (L)  (*post-mortem*) | - | - | - | Positive | <0.20^a^ | - | - | - | - | Negative^b^ | - | - | - | - | 55,000 ng (L) | - | - | [87] |
|  | 0.1-710 (L) (*post-mortem*) | - | - | - | - | - | - | - | - | - | - | - | - | - | - | - | - | - | [88] |
|  | - | - | - | - | 1432 (Ic) | - | - | - | - | - | - | - | - | - | - | - | - | - | [100] |
|  | <1-42.9 (L) (*post-mortem*) | - | - | - | - | - | - | - | - | - | - | - | - | - | - | - | - | - | [89] |
|  | - | - | - | - | 0.10-564.93 (Pm) | - | - | - | - | - | - | - | - | - | - | - | - | - | [134] |
|  | pBL: 0.95 (L) (*post-mortem*) | - | - | - | - | - | - | - | - | - | - | - | - | - | - | - | - | - | [105] |
| **Furanylfentanyl-dihydrodiol** | - | - | - | - | 0.1-500 (Pm) | - | - | - | - | - | - | - | - | - | - | - | - | - | [28] |
| **Furanylnorfentanyl** | - | - | - | - | 0.45-1.05 (Pm) | - | - | - | - | - | - | - | - | - | - | - | - | - | [28] |
| **Isobutyrylfentanyl** | 0.1-760 (L) (*post-mortem*) | - | - | - | - | - | - | - | - | - | - | - | - | - | - | - | - | - | [88] |
| **Isofentanyl or 3-methylbenzylfentanyl** | - | - | - | - | - | - | - | - | - | - | - | - | - | - | - | - | - | - | - |
| **Methoxyacetylfentanyl** | BL:20 (L)  fBL: 0.21-39.9 (L) (*post-mortem*) | - | - | - | - | - | - | - | - | - | - | - | - | - | - | - | - | - | [99] |
| **MT-45 or 1-cyclohexyl-4-(1,2-diphenylethyl)piperazine** | 6-157 (T) (ante-mortem) | - | - | - | - | - | - | - | - | - | - | - | - | - | - | - | - | - | [70] |
|  | pBL: 520 (L) (*post-mortem*) | - | - | - | - | - | - | - | - | - | - | - | - | - | - | - | - | - | [6] |
|  | pBL: 660 (L)  hBL: 1300 (L)  (*post-mortem*) | 1050 (L) | - | - | 370 (L) | 260 (L) | - | - | - | - | 24000 (L) | - | - | - | - | 49000 (L) | - | - | [52] |
|  | - | - | - | - | - | - | - | - | 1800-2000 (L) | 1500-1600 (L) | 3900-4100 (L) | 1500-1700 (L) | 8700-10900 (L) | - | - | - | - | - | [119] |
| **Norcarfentanil** | - | - | - | 0.2 (T) | 0.5 (T) | - | - | - | - | - | - | - | - | - | - | - | - | - | [113] |
|  | - | - | - | - | 1.70-28.90 (Pm) | - | - | - | - | - | - | - | - | - | - | - | - | - | [134] |
|  | 0.532 (L) (*post-mortem*) | - | - | - | - | 0.300 (L) | - | - | - | - | - | - | - | - | - | - | - | - | [115] |
| **Ocfentanil** | pBL: 15.3 (L)  hBL: 21.9-23.3 (L)  (*post-mortem*) | - | - | - | 6.0 (L) | 12.5 (L) | - | 13.7 (L) | - | 37.9 (L) | 31.2 (L) | 51.2 (L) | - | - | - | 17.1 (L) | - | 2999 ng/swab. (L) | [120] |
|  | pBL: 7.5-9.1 (L)  hBL: 27.9 (L)  (*post-mortem*) | - | - | - | 480 (L) | - | - | - | - | - | - | - | - | - | - | - | - | 360 (L) | [36] |
|  | pBL: 3.7 (L)  hBL: 3.9 (L) (*post-mortem*) | - | - | - | - | 2.0 (L) | - | 8.4 (L) | - | - | - | - | - | - | - | 2.5 (L) | - | - | [121] |
| **Ortho-fluorofentanyl** | 2.4 (L) (*post-mortem*) | - | - | 2.5 (T) | 3.9 (L) | - | - | - | - | - | - | - | - | - | - | - | - | - | [122] |
|  | 2.4 (L) (*post-mortem*) | - | - | - | - | - | - | - | - | - | - | - | - | - | - | - | - | - | [88] |
| **Para-chloroisobutyrylfentanyl or 4-chloroisobutyrylfentanyl or 4-chloroisobutylfentanyl** | 0.0081 µg/g (L) (*post-mortem*) | - | - | - | - | - | - | - | - | - | - | - | - | - | - | - | - | - | [44] |
|  | - | - | - | 5.1 (T) | - | - | - | - | - | - | - | - | - | - | - | - | - | - | [104] |
| **Para-fluorofentanyl or 4-fluorofentanyl** | 0.1-1 (L) (*post-mortem*) | - | - | - | - | - | - | - | - | - | - | - | - | - | - | - | - | - | [88] |
|  | - | - | - | - | 0.26-60.98 (Pm) | - | - | - | - | - | - | - | - | - | - | - | - | - | [134] |
| **Para-fluorobutyrfentanyl or Para-fluorobutyrylfentanyl or 4-fluorobutyrfentanyl or 4-fluorobutyrylfentanyl or Fluorobutyryl fentanyl** | - | - | - | 15.0 (T) | 9.5 (T) | - | - | - | - | - | - | - | - | - | - | - | - | - | [109] |
|  | 91-112 (L) (*post-mortem*) | - | - | - | 200-414 (L) | - | - | - | - | 248 (L) | 136-902 (L) | 197-411 (L) | - | - | - | 8450 ng/g (L) | - | - | [123] |
|  | 0.1-760 (L) (*post-mortem*) | - | - | - | - | - | - | - | - | - | - | - | - | - | - | - | - | - | [88] |
|  | - | - | - | - | 22-63 (Ic) | - | - | - | - | - | - | - | - | - | - | - | - | - | [100] |
|  | - | - | - | - | 0.11-693.00 (Pm) | - | - | - | - | - | - | - | - | - | - | - | - | - | [134] |
|  | 1.4 (L) (*post-mortem*) | - | - | - | - | - | - | - | - | - | - | - | - | - | - | - | - | - | [86] |
| **para-fluoroisobutyrfentanyl or para-fluoroisobutyrylfentanyl or 4-fluoroisobutyrfentanyl or 4-fluoroisobutyrylfentanyl** | - | - | - | 38 (L) | - | - | - | - | - | - | - | - | - | - | - | - | - | - | [104] |
|  | 0.026 µg/g (L) (*post-mortem*) | - | - | - | - | - | - | - | - | - | - | - | - | - | - | - | - | - | [44] |
|  | 0.1-760 (L) (*post-mortem*) | - | - | - | - | - | - | - | - | - | - | - | - | - | - | - | - | - | [88] |
|  | - | - | - | - | 134 (Ic) | - | - | - | - | - | - | - | - | - | - | - | - | - | [100] |
|  | 1.4 (L) (*post-mortem*) | - | - | - | - | - | - | - | - | - | - | - | - | - | - | - | - | - | [86] |
| **Remifentanil** | 0.0009 mg/kg (T) (ante-mortem) | - | - | - | - | - | - | - | - | - | - | - | - | - | - | - | - | - | [124] |
| **Sufentanil** | 1.1 (L) (*post-mortem*) | - | - | - | 1.3 (L) | 1.2 (L) | - | - | - | - | 1.75 (L) | 5.5 (L) | - | - | - | - | - | - | [125] |
|  | pBL: 27 (L)  hBL: 5.8 (L)  (*post-mortem*) | - | 0.2 (L) | - | 4.0 (L) | - | - | - | - | 0.3 (L) | 0.9 (L) | 0.8 (L) | 7.1 (L) | 2.1 (L) | - | - | - | - | [126] |
|  | 0.0028 mg/kg (T) (ante-mortem) | - | - | - | - | - | - | - | - | - | - | - | - | - | - | - | - | - | [124] |
|  | <0.4 (L) (*post-mortem*) | - | - | - | - | - | - | - | - | - | - | - | - | - | - | - | - | - | [89] |
| **Tetrahydrofuranylfentanyl or Tetrahydrofuranfentanyl** | - | - | - | 45 (T) | - | - | - | - | - | - | - | - | - | - | - | - | - | - | [104] |
|  | 339 (L) (*post-mortem*) | - | - | - | >5,000 (L) | - | - | - | - | - | - | - | - | - | - | - | - | - | [24] |
|  | - | - | - | - | 1942 (Ic) | - | - | - | - | - | - | - | - | - | - | - | - | - | [101] |
| **Thiofentanyl** | - | - | - | - | - | - | - | - | - | - | - | - | - | - | - | - | - | - | - |
| **U- 47700** | 13.8 (L) (*post-mortem*) | - | - | - | 71.0 (L) | - | - | - | - | - | - | - | - | - | - | - | - | - | [127] |
|  | BL: 453 (L)  pBL: 17-382 (L)  fBL: 217 (L)  aBL: 59-490 (L) (*post-mortem*) | - | - | - | - | - | - | - | - | - | - | - | - | - | - | - | - | - | [30] |
|  | pBL: 1460 (L)  (*post-mortem*) | - | - | - | - | - | - | - | - | - | - | - | - | - | - | - | - | - | [108] |
|  | - | - | - | 7.6 (T) | - | - | - | - | - | - | - | - | - | - | - | - | - | - | [59] |
|  | pBL: 525-819 (L)  hBL: 1,0.43-1,347 (L) (*post-mortem*) | - | - | - | 1,393-1,848 (L) | - | - | - | - | 97-110 (L) | 3,100-430 (L) | 140-270 (L) | 240-320 (L) | - | - | - | - | - | [128] |
|  | pBL: 190 (L)  hBL: 340 (L)  (*post-mortem*) | - | - | - | 360 (L) | 170 (L) | - | - | - | - | 1,700 (L) | - | - | - | - | <1 mg (L) | - | - | [125] |
|  | - | - | - | 240 (T) | - | - | - | - | - | - | - | - | - | - | - | - | - | - | [126] |
|  | pBL: 400 (L)  hBL: 260 (L)  (*post-mortem*) | - | - | - | 4600 (L) | 90 (L) | - | - | - | 380 (L) | 280 (L) | - | - | - | - | - | - | - | [62] |
|  | pBL: 360 (L)  (*post-mortem*) | - | - | - | - | - | - | - | - | - | - | - | - | - | - | - | - | - | [131] |
|  | - | - | - | - | 140-224 (T) | - | - | - | - | - | - | - | - | - | - | - | - | - | [132] |
|  | - | - | - | 228 (T) | 394 (T) | - | - | - | - | - | - | - | - | - | - | - | - | - | [133] |
|  | - | - | - | - | 0.1 (T) | - | - | - | - | - | - | - | - | - | - | - | - | - | [130] |
|  | 7.8-24 (L) (*post-mortem*) | - | - | - | - | - | - | - | - | - | - | - | - | - | - | - | - | - | [99] |
|  | 0.2-3800 (L) (*post-mortem*) | - | - | - | - | - | - | - | - | - | - | - | - | - | - | - | - | - | [88] |
|  | 17-453 (L) (*post-mortem*) | - | - | - | 48->1000 (L) | - | - | - | - | - | - | - | - | - | - | - | - | - | [65] |
|  | pBL: 330 (L) (*post-mortem*) | - | - | - | - | - | - | - | - | - | - | - | - | - | - | - | - | - | [131] |
|  | 74.5-547 (L) (*post-mortem*) | - | - | - | - | - | - | - | - | - | - | - | - | - | - | - | - | - | [89] |
|  | - | - | - | - | 370 (T) | - | - | - | - | - | - | - | - | - | - | - | - | - | [132] |
|  | - | - | - | - | 0.24-483.00 (Pm) | - | - | - | - | - | - | - | - | - | - | - | - | - | [134] |
|  | 1.85-2.19 (L)  iBL: 0.368 (L) (*post-mortem*) | - | - | - | - | - | - | - | - | - | - | - | - | - | - | 721 ng (L) | - | - | [133] |
| **U-49900** | 1.5 (L) (*post-mortem*) | - | - | - | 2.2 (L) | - | - | - | - | - | - | - | - | - | - | - | - | - | [24] |
| **U-50488** | - | - | - | - | - | - | - | - | - | - | - | - | - | - | - | - | - | - | - |
| **Valerylfentanyl** | 0.44 (L) (*post-mortem*) | - | - | - | - | - | - | - | - | - | - | - | - | - | - | - | - | - | [88] |

**Legend:** Blood (BL); Peripheral blood (pBL);; Cardiac/Central/Heart blood (hBL); Iliac blood (iBL); Abdominal Aorta blood (abBL); Aorta blood (aBL); Subclavian blood (sBL); Pericardial Fluid (PF); Cerebrospinal fluid (CF); Serum (S); Urine (U); Vitreous humor (VH); Hair (H); Bile (BI); Heart (H); Brain (BR); Liver (Ll); Kidney (K); Lung (LU); Muscle (M); Adipose tissue (AdT); Gastric contents (G); Spleen (SP); Nasal swabs (NS).

All concentrations are expressed in ng/mL except hair (pg/mg), Nasal swabs (ng) and tissues (ng/g) samples.

Information about the concentrations: (a) Confirmed results were below the limit of reporting (0.20 ng/mL); (b) Limit of reporting for liver was 0.40 ng/g; (DUID) Driving under the influence of drugs; (Ic) Intoxication cases; (L) Lethal concentration; (T) Toxic concentration; (Pm) Pain management.

**Note:** Furanylfentanyl-dihydrodiol and Furanylnorfentanyl are the metabolites of Furanylnorfentanyl; ANPP-SO_4_ is the metabolite of 4-ANPP; Norcarfentanil is the metabolite of Carfentanil; Despropionylmethylfentanyl is the metabolite of Alpha-methylfentanyl.

Concerning the anatomical site of sample collection, the terminology originally used by the authors was maintained.
